# Modifiable risk factors for inflammatory bowel disease in Kuwait: A cross-sectional analysis

**DOI:** 10.1371/journal.pone.0338005

**Published:** 2025-12-02

**Authors:** Reem Alhasan, Talal Alshatti

**Affiliations:** 1 Faculty of Public Health, Department of Environmental and Occupational Health, Kuwait University, Kuwait, Kuwait; 2 Faculty of Allied Health Sciences, Department of Physiotherapy, Kuwait University, Kuwait, Kuwait; PRISM CRO, PAKISTAN

## Abstract

Inflammatory bowel disease (IBD), a lifelong inflammatory condition of the gastrointestinal tract, is influenced by complex interactions between genetics and environmental exposures. While IBD rates have increased globally, largely due to Westernized lifestyle shifts, the contributors to IBD in Kuwait remain poorly understood. This cross-sectional study aimed to explore lifestyle, dietary, and occupational exposures associated with IBD in this unique population. A total of 520 adults (412 with IBD and 108 without IBD) completed a 40-item online questionnaire assessing early-life, lifestyle, occupational, and dietary exposures. Of the IBD participants, 174 had ulcerative colitis (UC) and 238 had Crohn’s disease (CD). The questionnaire was adapted from a previously validated tool, but was not formally validated in the Kuwaiti population. Associations between these factors and IBD status were analyzed using multivariable logistic regression with adjusted odds ratios (AORs) and 95% confidence intervals (CIs) reported after adjusting for potential confounders. Factors associated with increased odds of IBD included male sex (AOR = 2.487, p = 0.005), younger age (AOR = 0.964, p = 0.004), positive family history (AOR = 2.141, p = 0.023), mentally demanding jobs (AOR = 1.818, p = 0.039), alcohol consumption (AOR = 6.508, p = 0.016), frequent spicy food intake 1–2 times/week (AOR = 2.631, p = 0.008), and prior parasitic infection (AOR = 0.484, p = 0.042). A history of appendectomy was also associated (AOR = 7.158, p = 0.003). Several modifiable exposures were found to be associated with IBD status in Kuwait. These findings underscore the multifactorial nature of IBD. However, the cross-sectional design, reliance on self-reporting, and use of a non-validated instrument limit causal interpretation. Future longitudinal research is needed to better understand temporal relationships.

## Introduction

Inflammatory bowel disease (IBD) is a chronic, relapsing inflammatory condition of the gastrointestinal tract that poses a substantial health burden worldwide [1]. The two main types of IBD, Crohn’s disease (CD) and ulcerative colitis (UC), affect millions of individuals globally, with wide variability in incidence and prevalence across regions. This variability is influenced by factors such as geographic location, migration patterns, living environments, and ethnic backgrounds [2].

Recent epidemiological evidence indicates a shift in the global distribution of IBD. While incidence rates in historically high-burned regions such as North America and Europe have stabilized or even declined, cases are rising rapidly in newly industrialized nations. This trend is largely attributed to the adoption of Westernized lifestyle behaviors, including dietary changes and urban living, which are becoming more common in developing regions due to urbanization and globalization [3]. Europe currently reports incidence rates of up to 24.3 and 12.7 cases per 100,000 person-years for UC and CD, respectively, while regions such as the Middle East and Asia are witnessing a noticeable increase in new IBD diagnoses, reflecting the global spread of the disease [4].

The etiology of IBD is multifactorial, involving both genetic predispositions and environmental exposures. Although considerable progress has been made in identifying genetic variants associated with IBD, the partial penetrance of these genetic factors and increasing incidence rates in certain regions suggest that other influencing factors are key contributors to disease development. Factors such as diet, air pollution, antibiotic use, and psychological stress have been extensively studied; however, findings remain inconsistent or inconclusive [3,5]. This lack of clarity highlights the importance of investigating modifiable exposures potentially associated with IBD to better understand the etiology of IBD and develop preventive measures.

In Kuwait, IBD has emerged as a growing public health concern. A recent systematic review of 16 studies conducted across the Arab region—including Saudi Arabia, Kuwait, Egypt, Lebanon, and Oman—reported incidence rates of 2.33 per 100,000 for UC and 1.46 per 100,000 for CD, highlighting a rising trend of IBD across the region [6]. Despite this upward trend, research on IBD in Kuwait is scarce, with limited data on lifestyle factors contributing to its increasing prevalence. Most existing literature on IBD is based on studies conducted in Western populations, which may not fully reflect the unique exposures and cultural contexts present in Kuwait. This gap in the literature necessitates region-specific studies to better understand the factors influencing IBD development and guide targeted interventions.

To address the gap, the present cross-sectional study aimed to explore associations between a priori selected modifiable exposures—including lifestyle behaviors, dietary habits (spicy foods, frozen meals), lifestyle behaviors (sleep duration, physical activity, smoking), occupational factors (stressful or sedentary work), and early-life health factors (appendectomy, infections, antibiotic use) and the presence of IBD among adults in Kuwait. By identifying region-specific modifiable factors, this study aims to contribute to evidence-based public health interventions that may guide prevention strategies, mitigate IBD burden, and improve population health outcomes.

## Materials and methods

### Study design and study population

This study employed a cross-sectional design to examine associations between various exposures and IBD status among adults residing in Kuwait. Data were collected between November 2021 and May 2022 using a closed-ended online questionnaire consisting of dichotomous and multiple-choice items. These data have not been previously published and are being reported here for the first time. Although data collection was completed in 2022, analysis and reporting were conducted at a later stage due to extended data preparation, quality checks, and manuscript development. The survey targeted both individuals with IBD and healthy individuals without IBD to enable comparative analysis. Including healthy individuals allowed for the identification of exposure patterns among those without IBD, serving as a reference group. This comparative approach is essential in cross-sectional designs to estimate the strength and the direction of associations between environmental or lifestyle exposures and disease presence. It enhances the study’s ability to identify factors that may act as potential risks or protective elements in the Kuwaiti context.

Participants were eligible if they were ≥18 years old, of any nationality, and has resided in Kuwait for at least five years. Residence duration was confirmed by self-report, which was accepted due to the feasibility of online recruitment. IBD status was also self-reported, with participants identifying themselves as having received a diagnosis of CD or UC from a healthcare provider. However, this information was not verified through a review of medical records. Participants were also asked to report the year of their official diagnosis and the hospital where it was made. Despite its practicality for survey-based designs, this method introduces potential for misclassification, as medical record verification was not feasible. This is acknowledged as a limitation owing to the potential for recall or misclassification bias.

### Recruitment

A 40-item questionnaire was distributed through major social media platforms, including WhatsApp (via IBD support groups), X (formerly Twitter), Facebook, and Instagram, to recruit a diverse sample across Kuwait. Approximately 73% of IBD participants were recruited through patient support groups on WhatsApp, while the remaining IBD cases and most healthy participants were reached through broader social media circulation. This approach was selected due to Kuwait’s high internet penetration and wide geographic reach of these platforms. While cost-effective and efficient, the method may have introduced selection bias, which is acknowledged in the limitations.

Recruitment yielded a greater number of IBD respondents due to the active patient communities on social media. In Kuwait, IBD care is primarily provided in tertiary hospitals, with patients typically referred to gastroenterologists for diagnosis and management. Support groups for IBD patients are active online and serve as key engagement platforms for education. Recruiting healthy individuals without IBD was more challenging, contributing to the group imbalance.

### Sample size and power

The minimum required sample size was initially estimated using G*Power version 3.1, assuming a medium effect size (OR =1.5), α = 0.05, and 80% power of logistic regression. This indicated that at least 385 participants were needed. To account for potential incomplete responses, the target sample was increased to 400 [7].

To further ensure sufficient power for multivariable logistic regression analysis, the sample size was evaluated based on the events-per-variable (EPV) principle. We anticipated including up to 10–12 predictor variables in the final model. With 412 participants diagnosed with IBD, this yields an EPV > 34, well above the conventional threshold of 10 EPV, supporting the stability and reliability of the model estimates.

A total of 520 participants completed the questionnaire (412 with IBD and 108 without IBD), exceeding both the G*Power estimate and the EPV recommendation, thus strengthening the study’s statistical robustness.

### Data collection instrument

The questionnaire used in this study was adapted from a previously validated tool developed to assess lifestyle and dietary exposures in Yunnan Province, China [8]. Although the original version had undergone psychometric validation in a different setting, the adapted version used in this study was not formally validated for the Kuwaiti population due to resource and time constraints. Nevertheless, significant efforts were made to ensure the adapted questionnaire was culturally appropriate, comprehensible, and relevant to the local context.

To tailor the instrument to the Kuwaiti population, items related to diet, lifestyle behaviors, and occupational exposures were modified to reflect local terminology and common practices. The questionnaire was translated into Arabic using a forward–backward translation process and reviewed by bilingual public health professionals familiar with IBD research to ensure linguistic accuracy and conceptual consistency.

To enhance the tool’s face validity and usability, a pilot test was conducted with 20 individuals from the general population. Participants provided feedback on the clarity, structure, and relevance of the items. Based on this feedback, minor adjustments were made to item phrasing and response order to improve response flow. Although a full psychometric evaluation (e.g., construct validity, internal consistency) was not conducted, the combination of expert review, cultural adaptation, and pilot testing provides reasonable support for the instrument’s applicability in the current context. Future research should formally validate this adapted tool to strengthen its utility. No formal reliability metrics were collected, which is acknowledged as a limitation. Both the Arabic and the English versions of the questionnaire are provided in the supplementary information S1 and S2 Files.

### Data analysis

All data were analyzed using IBM SPSS Statistics version 26, the latest version available under our institutional license at the time of data analysis. Descriptive statistics were used to summarize participant demographics, health history, and exposure variables. Categorical variables were expressed as frequencies and percentages. Continuous variables were tested for normality and presented as medians due to non-normal distribution.

Chi-square tests were used to compare categorical variables between participants with IBD and those without IBD. A multivariable logistic regression analysis was conducted to assess the association between various exposures and IBD status. This method allowed for the adjustment of potential confounders and enabled the identification of independent risk and protective factors for IBD. Odds ratios (ORs) and 95% confidence intervals (CIs) were reported to quantify the strength of associations. The logistic regression model included pre-specified covariates such as age, sex, BMI, nationality, and years of residence. Exposure variables included occupational stress, dietary patters, sleep duration, smoking, alcohol use, and other environmental factors such as antibiotic/NSAID use, early life infections, breastfeeding history, and pet ownership. Ordinal and nominal variables were appropriately coded; ordinal items were treated as ordered categorical variables, and reference categories were selected based on the lowest risk groups. Missing data were minimal and handled using a complete-case approach. Multicollinearity was assessed using variance inflation factor (VIF), and model calibration was evaluated using the Hosmer-Lemeshow test. Discriminative ability of the model was assessed using the area under the receiver operating characteristic curve (AUC). Stepwise logistic regression models were developed to assess the impact of incremental covariate adjustment on selected exposure variables. These models included: (1) crude/unadjusted ORs, (2) ORs adjusted for sex, and (3) ORs adjusted for sex and age (Supplementary S5 Table). Additionally, a multivariable linear regression model was used to explore factors associated with age at diagnosis among IBD participants, with the goal of identifying exposures that may influence earlier disease onset.

The following exposure domains were evaluated in the logistic regression model:

1. Occupational stress and job type: Based on the hypothesis that chronic stress contributes to immune dysregulation and inflammation.
2. Dietary patterns: Including frequent intake of spicy foods, frozen meals, and irregular mealtimes—factors previously implicated in gut dysbiosis.
3. Sleep duration: Assessed due to its known role in immune function and chronic disease risk.
4. Physical activity: Included as a potential protective factor due to its anti-inflammatory benefits.
5. Smoking and alcohol consumption: Analyzed because of their established links to IBD pathogenesis.
6. Other environmental exposures: For example, appendectomy, antibiotic and NA-NSAID use, early-life infections, breastfeeding history, pet exposure, allergies, and parasitic infections.

### Ethical considerations

Participation was entirely voluntary, and electronic informed consent was obtained before accessing the questionnaire. Consent was integrated into the electronic questionnaire form and proceeding to the questionnaire indicated agreement. The questionnaire was anonymous and contained no personal identifiers. Confidentiality and data protection were maintained in accordance with ethical research guidelines.

This study was conducted in accordance with the ethical principles outlined in the Declaration of Helsinki. The study protocol was reviewed and approved by The Standing Committee for Coordination of Health and Medical Research – Ministry of Health, Kuwait [approval number: 1780/2021], and informed consent was obtained from all participants prior to participation. The study was reported in accordance with the STROBE guidelines for cross-sectional studies.

## Results

A total of 520 participants were included in the study, 174 with ulcerative colitis (UC), 238 with Crohn’s disease (CD), and 108 healthy non-IBD individuals. The majority of participants were females 66.0%, with a slightly higher proportion of females among non-IBD group 75.9% compared to UC 65.5%, and CD 61.8% groups. The mean age was similar across groups, ranging from 30.8 ± 9.5 years in the CD group to 33.6 ± 12.4 years in the non-IBD group. Median ages were 30, 30, 31 years, respectively. BMI was slightly higher in the non-IBD group (mean 26.4 ± 7.0 kg/m^2^) compared to UC (24.3 ± 4.9 kg/m^2^) and CD (24.3 ± 6.0 kg/m^2^). The vast majority of participants were of Middle Eastern nationality 87.7%, with small proportion identifying as American 3.1%, European 8.0%, Australian 0.4%, or Asian 0.8%. Nearly half the sample was single 49.6%, while 45.2% were married, and smaller proportions were divorced or widowed. Regarding occupation, half of the participants reported mixed work 50.0%, followed by mental work 41.7%, and manual work 8.3%. A detailed breakdown of demographic and clinical characteristics by group is shown in Table 1.

**Table 1:** Sociodemographic characteristics of study participants by disease status (UC, CD, and Non-IBD).

Characteristic	UC (n = 174)	CD (n = 238)	IBD Total (n = 412)	Non-IBD (n = 108)	Total (N = 520)
**Sex, n (%)**					
Female	114 (65.5%)	147 (61.8%)	261 (63.3%)	82 (75.9%)	343 (66.0%)
Male	60 (34.5%)	91 (38.2%)	151 (36.7%)	26 (24.1%)	177 (34.0%)
**Age (years)**					
Mean ± SD	32.9 ± 11.6	30.8 ± 9.5	31.66 ± 0.519	33.6 ± 12.4	
Median	30	30	30	31	
Min–Max	6–79	6–57	6-57	6–69	
**BMI (kg/m²)**					
Mean ± SD	24.3 ± 4.9	24.3 ± 6.0	24.3 ± 0.464	26.4 ± 7.0	
Median	24.2	23	23.53	24.6	
Min–Max	10.7–40.4	11.6–47.4	15.8-62.3	15.8–62.3	
**Nationality, n (%)**					
Middle Eastern	142 (81.6%)	206 (86.6%)	348 (84.5%)	108 (100%)	456 (87.7%)
American	7 (4.0%)	9 (3.8%)	16(3.9%)	0 (0.0%)	16 (3.1%)
European	22 (12.6%)	20 (8.4%)	42 (10.2%)	0 (0.0%)	42 (8.0%)
Australian	1 (0.6%)	1 (0.4%)	2 (0.5%)	0 (0.0%)	2 (0.4%)
Asian	2 (1.1%)	2 (0.8%)	4 (0.8%)	0 (0.0%)	4 (0.8%)
**Marital Status, n (%)**					
Single	83 (47.7%)	119 (50.0%)	202 (49.0%)	56 (51.9%)	258 (49.6%)
Married	83 (47.7%)	108 (45.4%)	191 (46.4%)	44 (40.7%)	235 (45.2%)
Divorced	6 (3.4%)	8 (3.4%)	14 (3.4%)	6 (5.6%)	20 (3.8%)
Widowed	2 (1.1%)	3 (1.3%)	5 (1.2%)	2 (1.9%)	7 (1.3%)
**Work Type, n (%)**					
Manual	13 (7.5%)	23 (9.7%)	36 (8.7%)	7 (6.5%)	43 (8.3%)
Mental	81 (46.6%)	106 (44.5%)	187 (45.4%)	30 (27.8%)	217 (41.7%)
Mixed	80 (46.0%)	109 (45.8%)	189 (45.9%)	71 (65.7%)	260 (50.0%)

Continuous variables are presented as mean ± standard deviation, median, and range (Min–Max). Categorical variables are presented as number (percentage). UC, ulcerative colitis; CD, Crohn’s disease; IBD, inflammatory bowel disease; BMI, body mass index.

### Chi-square tests

Chi-square tests were conducted to evaluate the relationship between various categorical variables and IBD status. The results both significant and non-significant are presented in Table 2. Several demographic, occupational, dietary, and early-life exposures showed statistically significant associations. These included occupational factors specifically work type (p = 0.006), with a greater proportion of individuals with IBD engaged in mental or mixed work roles. Dietary factors such as consumption of milk (p = 0.039), eating fried food (p = 0.046), eating spicy foods (p < 0.001), fish intake (p = 0.022), and frozen meal intake (p = 0.003) were also significantly associated with IBD. In terms of alcohol-related exposures, both alcohol consumption (p = 0.022) and type of alcohol consumed (p = 0.037) showed significant associations. Medical history variables were significantly related to IBD status included the presence of allergies (p = 0.005), history of appendectomy (p < 0.001), childhood antibiotic use before the age of 14 (p = 0.011), use of non-aspirin-non-steroidal anti-inflammatory drugs (NSAIDS) (borderline significant at p = 0.054), oral contraceptive use (p = 0.046), and prior parasitic infections (p = 0.020). all other exposures assessed did not demonstrate statistically significant associations.

**Table 2:** Chi-square test results for the association between risk factors and IBD status.

Variable		UC	CD	Non-IBD	Total	p-value
**Occupational Factors:**						
**Work type:**	*Manual work*	13 (7.50%)	23 (9.70%)	7(6.50%)	43 (8.30%)	0.006*
	*Mental work*	81 (46.60%)	106 (44.50%)	30 (27.80%)	217 (41.70%)	
	*Mixed work*	80 (46.60%)	109 (45.80%)	71 (65.70%)	260 (50.00%)	
**Work stress:**	*no stress*	17 (9.80%)	27 (11.30%)	11 (10.20%)	55 (10.60%)	0.161
	*mild stress*	25 (14.40%)	34 (14.30%)	8 (7.40%)	67 (12.90%)	
	*moderate stress*	77 (44.30%)	106 (44.50%)	39 (36.10%)	222 (42.70%)	
	*much stress*	44 (25.30%)	55 (23.10%)	37 (34.30%)	136 (26.20%)	
	*extreme stress*	11 (6.30%)	16 (6.70%)	13 (12.00%)	40 (7.70%)	
**Dietary patterns:**						
**Irregular mealtimes:**	*Never*	36 (20.70%)	45 (18.90%)	16 (14.80%)	97 (18.70%)	0.786
	*1-2 times/week*	60 (34.50%)	81 (34.00%)	41 (38.00%)	182 (35.00%)	
	*≥3 times/week*	78 (44.80%)	112 (47.10%)	51 (47.20%)	241 (46.30%)	
**Eating meat:**	*Never*	16 (9.20%)	33 (13.90%)	8 (7.40%)	57 (11.00%)	0.363
	*1-2 times/week*	75 (43.10%)	102 (43.00%)	48 (44.40%)	225 (43.40%)	
	*≥3 times/week*	83 (47.70%)	102 (43.00%)	52 (48.10%)	237 (45.70%)	
**Eating eggs:**	*Never*	18 (10.30%)	44 (18.50%)	19 (17.60%)	81 (15.60%)	0.091
	*1-2 times/week*	98 (56.30%)	112 (47.10%)	60 (55.60%)	270 (51.90%)	
	*≥3 times/week*	58 (33.30%)	82 (34.50%)	29 (26.90%)	169 (32.50%)	
**Consumption of milk:**	*Never*	70 (40.20%)	112 (47.10%)	32 (29.60%)	214 (41.20%)	0.039*
	*1-2 times/week*	49 (28.20%)	66 (27.70%)	39 (36.10%)	154 (29.60%)	
	*≥3 times/week*	55 (31.60%)	60 (25.20%)	37 (34.30%)	152 (29.20%)	
**Eating fried foods:**	*Never*	41 (23.60%)	54 (22.70%)	11 (10.20%)	106 (20.40%)	0.046*
	*1-2 times/week*	81 (46.60%)	102 (42.90%)	56 (51.90%)	239 (46.00%)	
	*≥3 times/week*	52 (29.90%)	82 (34.50%)	41 (38.00%)	175 (33.70%)	
**Eating salty foods (bacon, salted fish, pickled mustard green, etc.):**	*Never*	56 (32.20%)	76 (31.90%)	21 (19.40%)	153 (29.40%)	0.046*
	*1-2 times/week*	71 (40.80%)	92 (38.70%)	59 (54.60%)	222 (42.70%)	
	*≥3 times/week*	47 (27.00%)	70 (29.40%)	28 (25.90%)	145 (27.90%)	
**Eating spicy and or spiced foods:**	*Never*	72 (41.40%)	101 (42.40%)	18 (16.70%)	191 (36.70%)	<0.001*
	*1-2 times/week*	59 (33.90%)	70 (29.40%)	43 (39.80%)	172 (33.10%)	
	*≥3 times/week*	43 (24.70%)	67 (28.20%)	47 (43.50%)	157 (30.20%)	
**Consumption of sugars and sweets:**	*Never*	24 (13.80%)	32 (13.40%)	8 (7.40%)	64 (12.30%)	0.124
	*1-2 times/week*	78 (44.80%)	96 (40.30%)	39 (36.10%)	213 (41.00%)	
	*≥3 times/week*	72 (41.40%)	110 (46.20%)	61 (56.50%)	243 (46.70%)	
**Fish intake:**	*Never*	39 (22.40%)	85 (35.70%)	27 (25.00%)	151 (29.00%)	0.022*
	*1-2 times/week*	119 (68.40%)	133 (55.90%)	75 (69.40%)	327 (62.90%)	
	*≥3 times/week*	16 (9.20%)	20 (8.40%)	6 (5.60%)	42 (8.10%)	
**Frozen meals intake:**	*Never*	96 (55.20%)	125 (52.50%)	36 (33.30%)	257 (49.40%)	0.003*
	*1-2 times/week*	56 (32.20%)	91 (38.20%)	55 (50.90%)	202 (38.80%)	
	*≥3 times/week*	22 (1.60%)	22 (9.20%)	17 (15.70%)	61 (11.70%)	
**Vegetable intake:**	*Never*	21 (12.10%)	42 (17.60%)	14 (13.00%)	77 (14.80%)	0.246
	*1-2 times/week*	67 (38.50%)	102 (42.90%)	48 (44.40%)	217 (41.70%)	
	*≥3 times/week*	86 (49.40%)	94 (39.50%)	46 (42.60%)	226 (43.50%)	
**Consumption of fruits:**	*Never*	27 (15.50%)	38 (16.00%)	15 (13.90%)	80 (15.40%)	0.353
	*1-2 times/week*	77 (44.30%)	117 (49.20%)	61 (56.50%)	255 (49.00%)	
	*≥3 times/week*	70 (40.20%)	83 (34.90%)	32 (29.60%)	185 (35.60%)	
**Diet composition:**	*Vegetable-based*	10 (5.70%)	6 (2.50%)	4 (3.70%)	20 (3.80%)	0.152
	*Mixed meals*	162 (93.10%)	221 (92.90%)	101 (93.50%)	484 (93.10%)	
	*Meat-based*	2 (1.10%)	11 (4.60%)	3 (2.80%)	16 (3.10%)	
**Drinking water:**	*Tap water-based*	117 (67.20%)	136 (57.10%)	64 (59.30%)	317 (61.00%)	0.108
	*Boiled water-based*	1 (0.60%)	5 (2.10%)	0 (0.00%)	6 (1.20%)	
	*Mineral water-based*	56 (32.20%)	97 (40.80%)	44 (40.70%)	197 (37.90%)	
**Consumption of tea:**	*Yes*	98 (56.30%)	141 (59.20%)	69 (63.90%)	308 (59.20%)	0.454
	*No*	76 (43.70%)	97 (40.80%)	39 (36.10%)	212 (40.80%)	
**Frequency of tea consumption:**	*1-2 times/week*	26 (26.50%)	31 (22.00%)	26 (37.70%)	83 (26.90%)	0.055
	*≥3 times/week*	72 (73.50%)	110 (78.00%)	43 (62.30%)	225 (73.10%)	
**Main types of tea:**	*Black tea*	76 (77.60%)	111 (78.70%)	53 (78.80%)	240 (77.90%)	0.996
	*Green tea*	9 (9.20%)	12 (8.50%)	7 (10.10%)	28 (9.10%)	
	*Scented tea*	4 (4.10%)	4 (2.80%)	3 (4.30%)	11 (3.60%)	
	*Others*	9 (9.20%)	14 (9.90%)	6 (8.70%)	29 (9.40%)	
**Drinking Alcohol:**	*Yes*	15 (8.60%)	25 (10.50%)	2 (1.90%)	42 (8.10%)	0.022*
	*No*	159 (91.40%)	213 (89.50%)	106 (98.10%)	478 (91.90%)	
**Frequency of drinking:**	*1-2 times/month*	8 (53.30%)	10 (40.00%)	2 (100.00%)	20 (47.60%)	0.389
	*1-2 times/week*	5 (33.30%)	7 (28.00%)	0 (0.00%)	12 (28.60%)	
	*≥3 times/week*	2 (13.30%)	8 (32.00%)	0 (0.00%)	10 (23.8%)	
**Type of alcohol:**	*White wine*	0 (0.00%)	4 (16.00%)	0 (0.00%)	4 (9.50%)	0.037*
	*Red wine*	2 (13.30%)	3 (12.00%)	2 (100.00%)	7 (16.70%)	
	*Beer*	5 (33.30%)	8 (32.00%)	0 (0.00%)	13 (31.00%)	
	*high alcohol spirits*	8 (53.30%)	10 (40.00%)	0 (0.00%)	18 (42.90%)	
**Smoking:**	*Never smoking*	120 (69.00%)	165 (69.30%)	81 (75.00%)	366 (70.40%)	0.497
	*Smoking*	54 (31.00%)	73 (30.70%)	27 (25.00%)	154 (29.60%)	
**Type of smoking:**	*cigarettes*	33 (61.10%)	46 (63.00%)	11 (40.70%)	90 (58.40%)	0.345
	*vaping*	11 (20.40%)	16 (21.90%)	9 (33.30%)	36 (23.40%)	
	*hookah (shisha)*	10 (18.50%)	11 (15.10%)	7 (25.90%)	28 (18.20%)	
**Average number of cigarettes smoked or shisha (heads) per day:**	*<10 cigarettes*	30 (55.60%)	45 (61.60%)	16 (59.30%)	91 (59.10%)	0.589
	*10-20 cigarettes*	18 (33.30%)	24 (32.90%)	7 (25.90%)	49 (31.80%)	
	*>20 cigarettes*	6 (11.10%)	4 (5.50%)	4 (14.80%)	14 (9.10%)	
**If you are a current smoker or a former smoker – how long have you smoked?**	*<1year*	5 (9.30%)	7 (9.60%)	2 (7.40%)	14 (9.10%)	0.563
	*1-5 years*	11 (20.40%)	25 (34.20%)	7 (25.90%)	43 (27.90%)	
	*5-10 years*	12 (22.20%)	11 (15.10%)	3 (11.10%)	26 (16.90%)	
	*≥10 years*	26 (48.10%)	30 (41.10%)	15 (55.60%)	71 (46.10%)	
**Physical activity:**	*Never*	55 (31.60%)	85 (35.70%)	32 (29.60%)	172 (33.10%)	0.499
	*1-2 times/week*	77 (44.30%)	87 (36.60%)	44 (40.70%)	208 (40.00%)	
	*≥3 times/week*	42 (24.10%)	66 (27.70%)	32 (29.60%)	140 (26.90%)	
**Mean sleep duration:**	*<6 hours*	80 (46.00%)	106 (44.50%)	60 (55.60%)	246 (47.30%)	0.149
	*≥6 hours*	94 (54.00%)	132 (55.50%)	48 (44.40%)	274 (52.70%)	
**Family history**	*Yes*	38 (21.80%)	54 (22.70%)	16 (14.80%)	108 (20.80%)	0.225
	*No*	136 (78.20%)	184 (77.30%)	92 (85.20%)	412 (79.20%)	
**Allergies:**	*Yes*	41 (23.60%)	92 (38.70%)	34 (31.50%)	167 (32.10%)	0.005*
	*No*	133 (76.40%)	146 (61.30%)	74 (68.50%)	353 (67.90%)	
**Pet ownership:**	*Yes*	48 (27.60%)	60 (25.20%)	32 (29.60%)	140 (26.90%)	0.672
	*No*	126 (72.40%)	178 (74.80%)	76 (70.40%)	380 (73.10%)	
**Appendectomy:**	*Yes*	11 (6.30%)	46 (19.30%)	3 (2.80%)	60 (11.50%)	<0.001*
	*No*	163 (93.70%)	192 (80.70%)	105 (97.20%)	460 (85.50%)	
**Breast-feeding (I have been breast-fed before):**	*Never*	37 (21.30%)	45 (18.90%)	23 (21.30%)	105 (20.20%)	0.622
	*﹤3 months*	27 (15.50%)	35 (14.70%)	14 (13.00%)	76 (14.60%)	
	*≥3 months*	75 (43.10%)	10 (43.70%)	39 (36.10%)	218 (41.90%)	
	*Unsure*	3 (20.10%)	54 (22.70%)	32 (29.60%)	121 (23.30%)	
**Delivery mode:**	*Natural birth*	151 (86.80%)	200 (84.00%)	99 (91.70%)	450 (86.50%)	0.155
	*Cesarean*	23 (13.20%)	38 (16.00%)	9 (8.30%)	70 (13.50%)	
**Childhood antibiotic use (before 14 years):**	*Never*	41 (23.60%)	40 (16.80%)	30 (27.80%)	111 (21.30%)	0.011*
	*1-2 times/year*	45 (25.90%)	52 (21.80%)	22 (20.40%)	119 (22.90%)	
	*≥3 times/year*	24 (13.80%)	53 (22.30%)	9 (8.30%)	86 (16.50%)	
	*Unsure*	64 (36.80%)	93 (39.10%)	47 (43.50%)	204 (39.20%)	
**Childhood gastrointestinal infections (before 14 years):**	*Never*	98 (56.30%)	118 (49.60%)	67 (62.00%)	283 (54.40%)	0.098
	*1-2 times/year*	18 (10.30%)	26 (10.90%)	7 (6.50%)	51 (9.80%)	
	*≥3 times/year*	14 (8.00%)	34 (14.30%)	6 (5.60%)	54 (10.40%)	
	*Unsure*	44 (25.30%)	60 (25.20%)	28 (25.90%)	132 (25.40%)	
**Non-aspirin non-steroidal anti-inflammatory drugs (NA-NSAIDs) intake**	*Never*	98 (56.30%)	159 (66.80%)	73 (67.60%)	330 (63.50%)	0.054*
	*<1 month*	43 (24.70%)	54 (22.70%)	25 (23.10%)	122 (23.50%)	
	*≥1 month*	33 (19.00%)	25 (10.50%)	10 (9.30%)	68 (13.10%)	
**Aspirin intake:**	*Never*	147 (84.50%)	211 (88.70%)	95 (88.00%)	453 (87.10%)	0.316
	*<1 month*	20 (11.50%)	18 (7.60%)	6 (5.60%)	44 (8.50%)	
	*≥1 month*	7 (4.00%)	9 (3.80%)	7 (6.50%)	23 (4.40%)	
**Oral contraceptive use:**	*Never*	91 (52.60%)	148 (62.20%)	77 (71.30%)	316 (60.90%)	0.046*
	*Past*	27 (15.60%)	38 (16.00%)	14 (13.00%)	79 (15.20%)	
	*Current, < 5 years*	13 (7.50%)	7 (2.90%)	2 (1.90%)	22 (4.20%)	
	*Current, ≥ 5 years*	7 (4.00%)	8 (3.40%)	1 (0.90%)	16 (3.10%)	
	*Not applicable*	35 (20.20%)	37 (15.50%)	14 (13.00%)	86 (16.60%)	
**Parasitic infection:**	*Never*	119 (68.40%)	140 (58.80%)	86 (79.60%)	345 (66.30%)	0.002*
	*Past*	29 (16.70%)	43 (18.10%)	7 (6.50%)	79 (15.20%)	
	*Unsure*	26 (14.90%)	55 (23.10%)	15 (13.90%)	96 (18.50%)	

### Univariable logistic regression (unadjusted crude ratios)

Following chi-square analysis, variables showing potential associations with IBD status were further explored through univariable logistic regression to compute crude odds ratios (ORs) and 95% confidence intervals. This step aimed to assess the direction and the strength of association between each exposure and the likelihood of having IBD, without adjusting for confounding factors. Significant findings from this analysis revealed that several occupational, dietary, early-life exposure factors were associated with higher odds of IBD. the complete results are presented in Table 3. Variables that did not show significant associations in the crude model are available in the Supplementary Material (S3 Table) for reference.

**Table 3:** Crude odds ratios for factors associated with IBD (unadjusted univariable logistic regression).

Variable:	Reference Category	OR Exp(B)	95% C.I.for EXP(B)		p-value
			Lower	Upper	
***Sex:***	*Female*	0.548	0.338	0.89	0.015*
***Age:***		0.984	0.966	1.003	0.106
***Occupational Factors:***					
***Work type:***	*Manual*				0.001*
*Mental vs. manual*		1.932	0.822	4.54	0.091
*Mixed vs. manual*		2.342	1.46	3.755	p < 0.001
***Work Stress***	*No stress*				0.027*
*Mild stress*		1.926	0.756	4.906	0.169
*Moderate stress*		3.551	1.317	9.571	0.012*
*Much stress*		2.259	1.071	4.766	0.032*
*Extreme stress*		1.288	0.601	2.76	0.515
***Dietary Patterns:***					
***irregular mealtime***	*Never*	1.431			0.489
*1–2 times/week:*		1.359	0.732	2.523	0.331
* ≥ 3 times/week:*		0.923	0.58	1.47	0.736
***Meat intake***	*Never*				0.412
*1–2 times/week:*		1.722	0.767	3.863	0.188
* ≥ 3 times/week:*		1.036	0.665	1.614	0.874
***Eating salty foods***	*Never*				0.011*
*1–2 times/week:*		1.504	0.811	2.791	0.195
* ≥ 3 times/week:*		0.661	0.398	1.1	0.111
***Fish intake:***	*Never*				0.254
*1–2 times/week:*		0.765	0.293	1.998	0.585
* ≥ 3 times/week:*		0.56	0.227	1.38	0.208
***Consumption of sugar/sweets***	*Never*				0.048*
*1–2 times/week:*		2.346	1.059	5.198	0.036
* ≥ 3 times/week:*		1.495	0.951	2.351	0.081
***Consumption of milk***	*Never*				
*1–2 times/week:*		1.83	1.08	3.101	0.025*
* ≥ 3 times/week:*		0.949	0.565	1.594	0.842
***Eating fried foods***	*Never*				0.016*
*1–2 times/week:*		2.642	1.292	5.405	0.008*
* ≥ 3 times/week:*		1.313	0.631	1.584	0.244
***Eating spicy foods***	*Never*				p < 0.001*
*1–2 times/week:*		4.107	2.268	7.434	p < 0.001*
* ≥ 3 times/week:*		1.282	0.789	2.083	0.316
***Frozen meal intake***	*Never*				0.001*
*1–2 times/week:*		2.372	1.224	4.595	0.010*
* ≥ 3 times/week:*		1.033	0.545	1.958	0.922
***Vegetable intake:***	*Never*				0.755
*1–2 times/week:*		1.15	0.592	2.233	0.686
* ≥ 3 times/week:*		0.9	0.57	1.419	0.655
***Consumption of fruits***					0.215
*1–2 times/week:*	*Never*	0.906	0.46	1.786	0.776
* ≥ 3 times/week:*		0.665	0.413	1.072	0.094
***Diet composition***	*Vegetable-based*				0.975
*Mixed vs. vegetable-based*		0.923	0.174	4.885	0.925
*Meat vs. vegetable-based*		0.875	0.245	3.13	0.837
***Drinking Alcohol***	*Yes*	5.699	1.355	23.968	0.018*
***Smoking***	*Yes*	0.748	0.461	1.213	0.239
***Physical activity***	*Never*				0.645
*1–2 times/week:*		1.296	0.747	2.248	0.356
* ≥ 3 times/week:*		1.104	0.659	1.85	0.706
***Family history***	*Yes*	1.653	0.926	2.951	0.089
***Allergies***	*Yes*	1.038	0.658	1.636	0.874
***Pet ownership***	*Yes*	0.844	0.529	1.347	0.477
***Breastfeeding***	*Never*				0.287
* < 3 months*		1.282	0.694	2.369	0.428
* ≥ 3 months*		1.592	0.785	3.228	0.197
*Unsure*		1.65	0.969	2.81	0.065
***Delivery mode***	*Natural*	0.523	0.251	1.09	0.084
***Mean sleep duration:***	*<6 hours*	0.658	0.43	1.008	0.055*
***Appendectomy***	*Yes*	5.62	1.725	18.311	0.004*
***Childhood antibiotic use (before 14 years)***	*Never*				0.033*
*1–2 times/year*		1.32	0.749	2.325	0.337
* ≥ 3 times/year*		2.561	1.94	5.496	0.016*
***Childhood gastrointestinal infections (before 14 years):***	*Never*				0.121
*1–2 times/year*		0.868	0.527	1.43	0.578
* ≥ 3 times/year*		1.692	0.688	4.163	0.252
*Unsure*		2.154	0.837	5.545	0.112
***Non-aspirin non-steroidal anti-inflammatory drugs (NA-NSAIDs) intake***	*Never*				0.394
< 1 month		0.607	0.296	1.247	0.174
≥ 1 month		0.669	0.3	1.492	0.326
***Aspirin intake***	*Never*				0.271
* < 1 month*		1.649	0.659	4.123	0.285
* ≥ 1 month*		2.771	0.804	9.546	0.106
***Oral contraceptive use***	*Never*				0.121
*Past*		0.604	0.322	1.13	0.115
*Current, < 5years*		0.903	0.4	2.036	0.805
*CURRENT, ≥ 5years*		1.944	0.408	9.274	0.404
*Not applicable*		2.917	0.356	23.905	0.319
**Parasitic infection**	*Never*				0.004*
*Past*		0.558	0.305	1.019	0.058
*Unsure*		1.905	0.735	4.933	0.184

Odds ratios are unadjusted and reflect the crude association between each variable and IBD status without controlling for covariates.

### Multivariable logistic regression analysis

To account for confounding variables, multivariable logistic regression was conducted. Table 4. presents Adjusted Odds Ratios (AORs) and 95% CI. In the adjusted model, several variables showed significant associations with IBD. Males had 2.487 higher odds of IBD compared to females (AOR = 2.487; 95% CI: 1.315–4.704; p = 0.005), and increasing age was associated with a slightly reduced odds of IBD (AOR = 0.964 per year; 95% CI: 0.940–0.988; p = 0.004).

**Table 4:** Adjusted ORs and p-values for factors associated with IBD in the multivariable logistic regression model.

Variable	Reference Category	Adjusted OR	95% CI	p-value
Sex	Male vs. Female (ref)	2.487	1.315–4.704	0.005*
Age	Per 1 year increase	0.964	0.940–0.988	0.004*
Work type				
	Mental vs Manual (ref)	1.818	1.032–3.204	0.039*
	Mixed vs Manual (ref)	1.362	0.467–3.974	0.572
Work stress				
	Mild vs No stress (ref)	1.488	0.456–4.857	0.511
	Moderate vs No stress (ref)	1.811	0.552–5.936	0.327
	Severe vs No stress (ref)	1.565	0.609–4.020	0.353
Dietary patterns:				
Consumption of milk				
	1–2x/week vs Never (ref)	1.557	0.816–2.972	0.188
	≥3x/week vs Never (ref)	1.216	0.656–2.255	0.535
Eating fried foods				
	1–2x/week vs Never (ref)	1.283	0.516–3.189	0.591
	≥3x/week vs Never (ref)	0.747	0.415–1.344	0.333
Eating spicy foods				
	1–2x/week vs Never (ref)	2.631	1.287–5.378	0.008*
	≥3x/week vs Never (ref)	1.054	0.585–1.900	0.866
Consumption of sugar/sweets				
	1–2x/week vs Never (ref)	1.508	0.552–4.116	0.423
	≥3x/week vs Never (ref)	1.688	0.973–2.928	0.062
Frozen meal intake				
	1–2x/week vs Never (ref)	1.686	0.751–3.787	0.206
	≥3x/week vs Never (ref)	0.904	0.420–1.946	0.797
Drinking alcohol	Yes vs No (ref)	6.508	1.418–29.863	0.016*
Mean sleep duration	>6h vs < 6h (ref)	0.727	0.437–1.209	0.219
Family history	Yes vs No (ref)	2.141	1.109–4.134	0.023*
Appendectomy	Yes vs No (ref)	7.158	3.095–16.582	0.003*
Delivery mode	Cesarean vs Natural birth (ref)	0.716	0.309–1.658	0.435
Oral contraceptive use				
	Past vs Never (ref)	0.692	0.320–1.493	0.343
	Current < 5y vs Never (ref)	1.784	0.630–5.051	0.275
	Current ≥ 5y vs Never (ref)	3.185	0.658–22.683	0.135
	Not applicable vs Never (ref)	8.187	0.834–80.323	0.071
Parasitic infection				
	Past vs Never (ref)	0.484	0.240–0.973	0.042*
	Current vs Never (ref)	1.748	0.609–5.021	0.304

AORs represent adjusted odds ratios from the multivariable logistic regression model controlling for all variables listed. CI = confidence interval; p-value <0.05 indicate statistical significance.

Participants with mentally demanding work had higher odds of IBD than those in manual labor (AOR = 1.818; 95% CI: 1.032–3.204; p = 0.039). Among dietary exposures, frequent spicy food consumption (1–2times/week) remained significantly associated with increased odds of IBD (AOR = 2.631; 95% CI: 1.287–5.378; p = 0.008).

Additional significant associations were observed for alcohol consumption (AOR = 6.508; 95% CI: 1.418–29.863; p = 0.016), positive family history of IBD (AOR = 2.141; 95% CI: 1.109–4.134; p = 0.023) and history of appendectomy (AOR = 7.158; 95% CI: 3.095–16.582; p = 0.003). Moreover, a past history of parasitic infection was associated with significantly lower odds of IBD compared to those who never had a parasitic infection (AOR = 0.484; 95% CI: 0.240–0.973; p = 0.042). Other exposures, including work stress, milk and fried food intake, frozen meals, oral contraceptive use, sleep duration, and delivery mode, did not reach statistical significance in the adjusted model. The direction and magnitude of associations remained generally consistent across stepwise models (Supplementary S5 Table), with notable changes observed after adjusting for sex and age.

**Fig 1:**
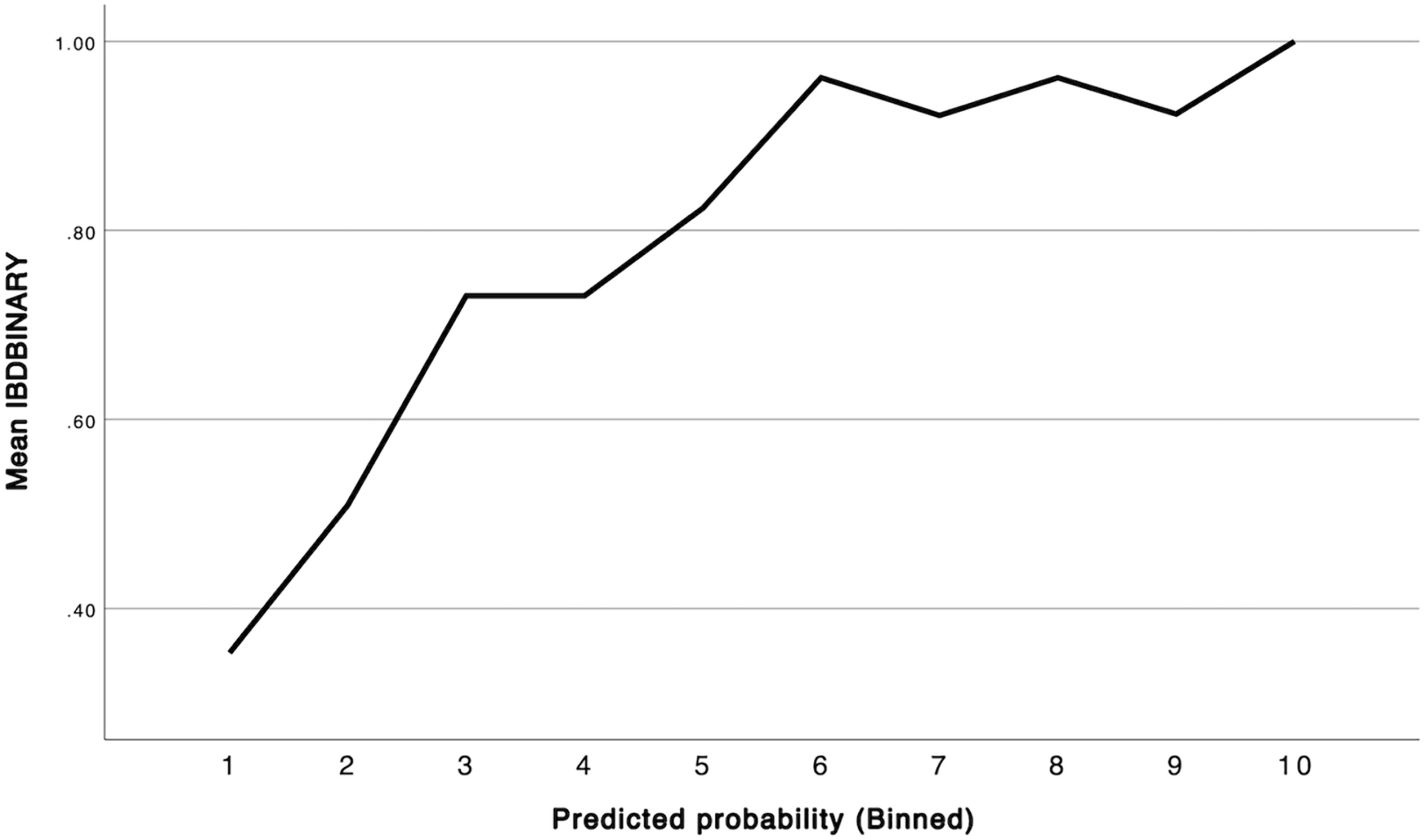
Calibration plot for the multivariable logistic regression model. The plot compares observed vs. predicted probabilities of IBD across ten deciles. The upward trend suggests good alignment between predicted risk and actual IBD occurrence.

**Fig 2:**
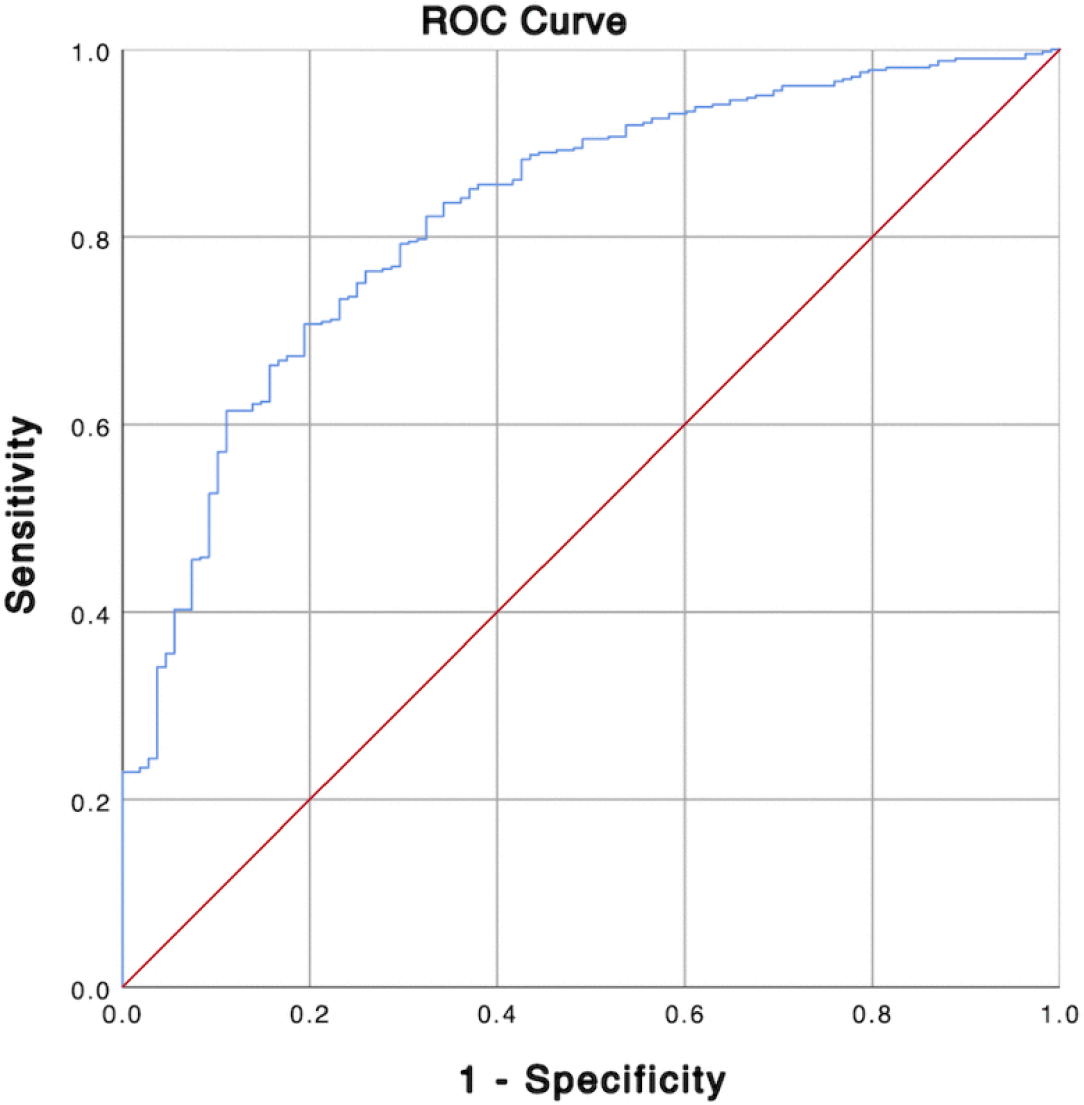
Receiver Operating Characteristic (ROC) curve for the final multivariable logistic regression model. The area under the curve (AUC) was 0.824 (95% CI: 0.782-0.867), indicating good model discrimination.

The final multivariable logistic regression model explained 33.1% of the variance in IBD status, as indicated by Nagelkerke R^2^ value. Model calibration was assessed using the Hosmer-Lemeshow goodness-of-fit test, which showed no evidence of poor fit (X^2 ^= 7.227, df = 8, p = 0.512). a calibration plot comparing observed and predicted probabilities across deciles of risk further confirmed adequate model fit (Fig 1) The model also demonstrated strong discriminative ability, with AUC of 0.824 (95% CI: 0.782–0.867, p < 0.001) as shown in the ROC curve (Fig 2). Multicollinearity diagnostics indicated no issues, with all variance inflation factor (VIF) values below 5.

### Interpretation of non-significant variables

Several variables, including work stress, frequent consumption of fried food, and milk and childhood antibiotic use, demonstrated significant associations in the univariable analysis but lost significance in the multivariable logistic regression. This attenuation may reflect confounding or shared variance with more robust predictors such as sex, age, dietary habits (e.g., spicy, and frozen meals and occupational exposures). These results reinforce the importance of multivariable adjustments when examining complex health outcomes like IBD, where multiple interrelated factors may obscure independent effects.

## Discussion

This cross-sectional study investigated the associations between demographic, dietary, occupational, and lifestyle factors and IBD in a sample of individuals living in Kuwait. The multivariable logistic regression revealed significant associations with sex, age, occupational exposure (work type), dietary habits, e.g., eating spicy food, drinking alcohol), family history, appendectomy, and parasitic infection. While these factors showed statistically significant associations with IBD status, the cross-sectional design limits causal interpretation. The findings largely contribute to the growing body of literature exploring environmental and lifestyle correlates of IBD but should be interpreted as associative rather than predictive.

In our study, male sex was significantly associated with higher odds of having inflammatory bowel disease (AOR: 2.84, p = 0.005), suggesting that males were nearly three times more likely to be diagnosed with IBD compared to females. This finding aligns with the recent regional analysis demonstrating a higher incidence and burden of IBD among males in the Middle East and North Africa (MENA) region, particularly for CD [9]. The authors noted a steady increase in the male-to-female ratio over the past two decades, possibly driven by sex-specific environmental exposures, differential immune responses, and healthcare-seeking behaviors in the region. While globally the sex distribution of IBD varies, with UC more common among males in some populations and CD more prevalent among females in others, the regional patterns observed in our sample echo the shifting epidemiology described in Middle Eastern cohorts. Cultural factors, occupational exposures, and access to care may further contribute to this observed disparity. Regarding age, our analysis found that with every one-year increase in age, the odds of having IBD decreased slightly (AOR = 0.964), p = 0.004), indicating that younger individuals are more likely to be affected. This is consistent with the global epidemiology of IBD, which shows peak onset typically occurs in the second and third decades of life [10]. In the Middle east region specifically, the 20–44 year age group carries the highest burden, highlighting the impact of IBD on young adults during their most productive years [9]. This age-related trend may reflect the role of early-life exposure, shifting lifestyles, and westernized dietary patterns.

In our analysis, participants engaged in mentally demanding occupations had significantly higher odds of IBD compared to those in manual labor roles (AOR = 1.818, p = 0.039). This finding contrasts with a recent case-control study which found no clear association between occupational category and IBD [11]. This discrepancy may reflect contextual differences unique to the Kuwaiti labor market, where manual work is often associated with lower socioeconomic status, but also with higher levels of physical exertion and limited sedentary time—factors that may confer protective effects. In contrast, mentally demanding jobs may involve in chronic psychological stress [12], prolonged sedentary behavior, and potential disruption of circadian rhythms [13,14], all of which have been implicated in IBD pathogenesis. Additionally, environmental exposures and work-related stress may differ substantially between occupational categories. These findings highlight the need for further research into occupational exposures as potentially modifiable risk factor for IBD, especially in region-specific and sociocultural contexts.

In our study, alcohol consumption was significantly associated with increased odds of having IBD (AOR = 6.508, p = 0.016), a finding supported by mechanistic and clinical evidence. Chronic alcohol use is known to disrupt the gut barrier and promote systemic inflammation by enhancing the translocation of gut-derived lipopolysaccharides (LPS) into the bloodstream. These microbial products stimulate hepatic and systemic inflammatory responses and contribute to multi-organ dysfunction, including the gut-liver-brain-axis, potentially exacerbating intestinal inflammation seen in IBD [15]. Similarly, spicy food consumption 1–2times/week was also found to be significantly associated with IBD risk (AOR = 2.631, p = 0.008). While some evidence suggests that capsaicin (the active compound in chili) may have anti-inflammatory effects in certain contexts, epidemiological findings remain mixed. For example, a recent population-based study from China observed that frequent spicy food intake was associated with higher risks of metabolic disorders such as obesity, dyslipidemia, and hypertension conditions that may share common inflammatory pathways with IBD [16]. The observed association in our study may reflect the role of spicy foods in triggering gastrointestinal symptoms, altering gut microbiota, or enhancing mucosal permeability, all of which could contribute to IBD pathogenesis in susceptible individuals. Mechanistic evidence from animal studies further supports this hypothesis; for instance, research on dietary supplementation in dairy cows demonstrated that specific nutrient exposures significantly altered hind-gut microbiota composition and nitrogen metabolism, emphasizing the role of diet in modulating gut microbial environments [17]. In addition, nutrient regulation has been shown to affect macrophage-controlled mucosal healing, a process central to intestinal immune balance [18]. Further research is warranted to explore these dietary-immune interactions in diverse populations.

Additionally, environmental contaminants have been shown to disrupt metabolic and inflammatory pathways relevant to intestinal health, providing broader biological plausibility for diet-related findings in IBD [19,20].

In our study, a positive family history was independently associated with more than double the odds of IBD (AOR = 2.141, p = 0.023), underscoring inherited susceptibility in disease risk. Familial aggregation is well documented: individual with a first-degree relative affected by IBD carry substantially elevated risk compared to the general population [21,22]. Moreover, a multicenter pediatric study from Saudi Arabia found that having a family history of IBD significantly increased risk of childhood disease, especially among first-degree relatives [23]. Although the effect size in our study is more modest than some reports (which report 4–8-fold increase), it remains meaningful considering adjustment for confounders and suggests that genetic and shared environmental factors contribute significantly to IBD susceptibility in our setting.

A history of appendectomy was significantly associated with IBD in our study (AOR = 7.158, p = 0.003), suggesting a potentially important clinical correlate in this population. While this finding aligns with prior studies that have reported positive associations and IBD incidence [24], the direction and strength of this relationship are known to vary by disease subtype. Specifically, appendectomy has been frequently reported to exert a protective effect against UC but may increase susceptibility to CD or exert a neutral effect, depending on the timing and indication for surgery [25–27]. In our data, appendectomy was reported by 6.3% of UC cases, 19.3% of CD cases, and 2.8% of non-IBD participants, and this difference was highly significant (χ2 = 26.9, p < 0.001). These descriptive results suggest that the strong overall positive association observed in the pooled IBD model is likely driven primarily by CD cases, consistent with literature reporting increased CD risk following appendectomy, particularly when performed before disease onset. Because our regression model aggregated UC and CD participants (n = 174, and n = 238, respectively), the subtype-specific effects may have been masked in the pooled analysis. This finding underscores the importance of considering disease heterogeneity and surgical timing when interpreting appendectomy-IBD associations. Future investigations in Kuwait should stratify analyses by IBD subtype and incorporate data on the age and indication for appendectomy, to clarify whether the observed effect reflects CD-specific susceptibility or broader immune-modulating mechanisms. Such differentiation would refine risk interpretation and support more nuanced public health messaging regarding appendectomy history and IBD epidemiology.

One of the most compelling findings was the negative association between parasitic infection and IBD (AOR = 0.484, p = 0.042). Lending support to the hygiene hypothesis, which suggests that reduced microbial exposure, common in highly urbanized settings, may impair immune system development and increase susceptibility to immune-mediated diseases such as IBD [28]. Innate antimicrobial peptides play a key role in maintaining host-microbe balance, providing a biological explanation for the protective association observed with parasitic exposure [29]. In Kuwait, where sanitation standards have improved dramatically over recent decades, this observation may reflect shifting microbial exposures due to rapid urbanization and public health infrastructure improvements.

Several variables such as fried food consumption, milk intake, work-related stress, and frequent childhood antibiotic use were significantly associated with IBD in the crude unadjusted analyses, but lost significance after multivariable adjustment. This attenuation may suggest confounding by stronger predictors included in the model. For instance, while moderate work stress was associated with increased IBD odds in the crude model (OR=3.551, p = 0.012), this effect diminished after adjustment, whereas mentally demanding work remained significant (AOR = 1.818, p = 0.039). this may imply that the specific classification of “mental work” captures a more chronic or biologically relevant component of occupational strain (e.g., cognitive overload, circadian disruption) than the broader and potentially more subjective construct of perceived stress. Similarly, childhood antibiotic use (≥3times/year) was associated with IBD in unadjusted analysis (OR=2.561, p = 0.016), but this association may be confounded by age or overlapping exposures such as early-life infections, which were included as covariates. These patterns underscore the multifactorial nature of IBD in Kuwait, where both traditional (e.g., genetics, age) and transitional (e.g., occupational, dietary) risk factors intersect. This multidimensional pattern aligns with broader evidence highlighting interactions between intestinal and systemic pathways often conceptualized through gut-organ regulatory axes which may influence immune activity and metabolic homeostasis [30]. Tailored public health strategies targeting robust, modifiable exposures may offer promising avenues for prevention and control in the region.

### Strengths and limitations

This study is among the first in Kuwait to explore a wide range of environmental, occupational, dietary, and lifestyle factors associated with IBD, contributing region-specific evidence to a globally growing body of literature. Key strengths include a relatively large sample size (n = 520), the application of a multivariable logistic regression to reduce confounding, and the use of a culturally tailored questionnaire. The instrument underwent forward–backward translation, expert review, and pilot testing to ensure contextual relevance and content clarity to the Kuwaiti population. In addition, recruitment through social media enabled broad geographic reach across Kuwait and inclusion of long-term residents from diverse backgrounds. Together, these strengths support the internal validity and contextual relevance of the findings.

Despite these strengths, several limitations must be acknowledged. First, the non-random, convenience-based sampling strategy introduces a high risk of selection bias, limiting generalizability of results to wider Kuwaiti population. The sample included a disproportionate number of participants with IBD (n = 412) compared to those without (n = 108), and 70% of IBD respondents were recruited through online patient support groups. Such participants may represent individuals with more severe, chronic, or earlier-onset disease, who are more engaged in health advocacy or online communities. Consequently, they may recall exposures differently or have made greater lifestyle and dietary changes following diagnosis, potentially inflating associations between certain modifiable exposures and IBD status. While regression model adjusted for covariates to mitigate confounding, the underlying imbalance may still bias effect estimates toward the experiences of digitally engaged and health-conscious individuals.

Second, while the questionnaire was adapted from a validated tool originally developed in Yunnan Province, China, it was not formally psychometrically validated in the Kuwaiti context due to time and resource constraints. Although steps were taken to ensure cultural relevance and clarity, including expert review, translation, and pilot testing, the lack of formal validation limits the instrument’s construct validity and reliability.

Third, all data were self-reported, including disease diagnosis, exposure history, and health behaviors. IBD status was not verified through medical records or clinical confirmation, increasing the risk of misclassification bias. This limitation affects both the accuracy of disease categorization and the recall of past exposures, such as early-life factors or dietary habits. Future studies may benefit from incorporating objective, technology-aided diagnostic tools to reduce misclassification risk and enhance diagnostic precision. Recent advancements in Artificial Intelligence, such as deep learning-based models for UC evaluation, demonstrate the potential of automated-fine grained phenotyping to support more accurate and scalable data collection in IBD research [31].

Fourth, the recruitment method via social media platforms (e.g., WhatsApp, Instagram, X, and Facebook) may have introduced selection bias, potentially overrepresenting younger, more urban, and technologically connected individuals while underrepresenting older or less digitally engaged populations. These challenges are common in digital survey-based research and highlight the importance of developing stronger digital health infrastructure to support high-quality, representative data collection. Evidence from recent research underscores that integrated regional health systems—particularly those fostering innovation in digital health technologies—can significantly enhance the accuracy and equity of epidemiological surveillance across local and national contexts [32].

Lastly, the cross-sectional nature of this study limits the ability to infer causality, as both exposure and outcome were assessed at the same point in time. While associations between exposures and IBD were identified, longitudinal studies are required to establish temporality and determine whether these exposures contribute casually to disease onset or progression. Future longitudinal studies should aim to test targeted hypotheses—for instance, whether modifiable exposures such as dietary patterns, alcohol consumption, smoking, or occupational stress independently predict IBD onset or exacerbate disease progression in genetically predisposed individuals.

## Conclusions

This cross sectional study is the first in Kuwait to examine several modifiable lifestyle, medical and occupational factors associated with IBD. The study identified several factors associated with increased odds of IBD among adults in Kuwait, including sex, age, family history, work type, appendectomy, alcohol consumption, spicy food intake, and parasitic infection. These findings reinforce the multifactorial nature of IBD and highlight the interplay between genetic predisposition, lifestyle exposures, and environmental influences. While the cross-sectional design limit causal inference, the results offer valuable insights for region-specific public health strategies and risk-reduction efforts. Further research and continued exploration of modifiable exposures in Middle East populations is warranted to inform prevention-oriented approaches and targeted health education initiatives.

## Supporting information

S1 Table Sociodemographic characteristics of study participants by disease status (UC, CD, IBD total, and non IBD group). (DOCX)

S2 Table Chi-square test results for the association between risk factors and IBD status. (DOCX)

S3 Table Crude odds ratios for factors associated with IBD (unadjusted univariable logistic regression). (DOCX)

S4 Table Adjusted odds ratios and p-values for factors associated with IBD in the multivariable logistic regression model. (DOCX)

S5 Table Stepwise logistic regression model showing the impact of covariate adjustment on associations between selected exposures and IBD status. (DOCX)

S1 File Questionnaire. English version of the questionnaire assessing environmental and lifestyle factors associated with IBD.
(DOCX)

S2 File Questionnaire. Arabic version of the questionnaire assessing environmental and lifestyle factors associated with IBD.
(DOCX)
