# Supplementary material for: Modifiable risk factors for inflammatory bowel disease in Kuwait: A cross-sectional analysis
Source: PLoS One. 2025 Dec 2;20(12):e0338005. doi: 10.1371/journal.pone.0338005 (PMC12671769; doi:10.1371/journal.pone.0338005)
Supplement: S1 Table — (DOCX) [file pone.0338005.s001.docx]

**Table 1.: Sociodemographic characteristics of study participants by disease status (UC, CD, and Non-IBD)**

| ***Characteristic*** | ***UC (n=174)*** | ***CD (n=238)*** | ***IBD Total***  ***(n=412)*** | ***Non-IBD (n=108)*** | ***Total (N=520)*** |
| --- | --- | --- | --- | --- | --- |
| ***Sex, n (%)*** |  |  |  |  |  |
| *Female* | *114 (65.5%)* | *147 (61.8%)* | *261 (63.3%)* | *82 (75.9%)* | *343 (66.0%)* |
| *Male* | *60 (34.5%)* | *91 (38.2%)* | *151 (36.7%)* | *26 (24.1%)* | *177 (34.0%)* |
| ***Age (years)*** |  |  |  |  |  |
| *Mean ± SD* | *32.9 ± 11.6* | *30.8 ± 9.5* | *31.66± 0.519* | *33.6 ± 12.4* |  |
| *Median* | *30* | *30* | *30* | *31* |  |
| *Min–Max* | *6–79* | *6–57* | *6-57* | *6–69* |  |
| ***BMI (kg/m²)*** |  |  |  |  |  |
| *Mean ± SD* | *24.3 ± 4.9* | *24.3 ± 6.0* | *24.3 ± 0.464* | *26.4 ± 7.0* |  |
| *Median* | *24.2* | *23* | *23.53* | *24.6* |  |
| *Min–Max* | *10.7–40.4* | *11.6–47.4* | *15.8-62.3* | *15.8–62.3* |  |
| ***Nationality, n (%)*** |  |  |  |  |  |
| *Middle Eastern* | *142 (81.6%)* | *206 (86.6%)* | *348 (84.5%)* | *108 (100%)* | *456 (87.7%)* |
| *American* | *7 (4.0%)* | *9 (3.8%)* | *16(3.9%)* | *0 (0.0%)* | *16 (3.1%)* |
| *European* | *22 (12.6%)* | *20 (8.4%)* | *42 (10.2%)* | *0 (0.0%)* | *42 (8.0%)* |
| *Australian* | *1 (0.6%)* | *1 (0.4%)* | *2 (0.5%)* | *0 (0.0%)* | *2 (0.4%)* |
| *Asian* | *2 (1.1%)* | *2 (0.8%)* | *4 (0.8%)* | *0 (0.0%)* | *4 (0.8%)* |
| ***Marital Status, n (%)*** |  |  |  |  |  |
| *Single* | *83 (47.7%)* | *119 (50.0%)* | *202 (49.0%)* | *56 (51.9%)* | *258 (49.6%)* |
| *Married* | *83 (47.7%)* | *108 (45.4%)* | *191 (46.4%)* | *44 (40.7%)* | *235 (45.2%)* |
| *Divorced* | *6 (3.4%)* | *8 (3.4%)* | *14 (3.4%)* | *6 (5.6%)* | *20 (3.8%)* |
| *Widowed* | *2 (1.1%)* | *3 (1.3%)* | *5 (1.2%)* | *2 (1.9%)* | *7 (1.3%)* |
| ***Work Type, n (%)*** |  |  |  |  |  |
| *Manual* | *13 (7.5%)* | *23 (9.7%)* | *36 (8.7%)* | *7 (6.5%)* | *43 (8.3%)* |
| *Mental* | *81 (46.6%)* | *106 (44.5%)* | *187 (45.4%)* | *30 (27.8%)* | *217 (41.7%)* |
| *Mixed* | *80 (46.0%)* | *109 (45.8%)* | *189 (45.9%)* | *71 (65.7%)* | *260 (50.0%)* |
