# Supplementary material for: Modifiable risk factors for inflammatory bowel disease in Kuwait: A cross-sectional analysis
Source: PLoS One. 2025 Dec 2;20(12):e0338005. doi: 10.1371/journal.pone.0338005 (PMC12671769; doi:10.1371/journal.pone.0338005)
Supplement: S2 Table — (DOCX) [file pone.0338005.s002.docx]

**Table 2. Chi-square test results for the association between risk factors and IBD status**

| **Variable** |  |  | **UC** | **CD** | **Non-IBD** | **Total** | **p-value** |
| --- | --- | --- | --- | --- | --- | --- | --- |
| Occupational Factors: |  |  |  |  |  |  |  |
| **Work type:** | *Manual work* |  | 13 (7.50%) | 23 (9.70%) | 7(6.50%) | 43 (8.30%) | 0.006* |
|  | *Mental work* |  | 81 (46.60%) | 106 (44.50%) | 30 (27.80%) | 217 (41.70%) |  |
|  | *Mixed work* |  | 80 (46.60%) | 109 (45.80%) | 71 (65.70%) | 260 (50.00%) |  |
| **Work stress:** | *no stress* |  | 17 (9.80%) | 27 (11.30%) | 11 (10.20%) | 55 (10.60%) | 0.161 |
|  | *mild stress* |  | 25 (14.40%) | 34 (14.30%) | 8 (7.40%) | 67 (12.90%) |  |
|  | *moderate stress* |  | 77 (44.30%) | 106 (44.50%) | 39 (36.10%) | 222 (42.70%) |  |
|  | *much stress* |  | 44 (25.30%) | 55 (23.10%) | 37 (34.30%) | 136 (26.20%) |  |
|  | *extreme stress* |  | 11 (6.30%) | 16 (6.70%) | 13 (12.00%) | 40 (7.70%) |  |
| **Dietary patterns:** |  |  |  |  |  |  |  |
| **Irregular mealtimes:** | *Never* |  | 36 (20.70%) | 45 (18.90%) | 16 (14.80%) | 97 (18.70%) | 0.786 |
|  | *1-2 times /week* |  | 60 (34.50%) | 81 (34.00%) | 41 (38.00%) | 182 (35.00%) |  |
|  | *≥3 times /week* |  | 78 (44.80%) | 112 (47.10%) | 51 (47.20%) | 241 (46.30%) |  |
| **Eating meat:** | *Never* |  | 16 (9.20%) | 33 (13.90%) | 8 (7.40%) | 57 (11.00%) | 0.363 |
|  | *1-2 times /week* |  | 75 (43.10%) | 102 (43.00%) | 48 (44.40%) | 225 (43.40%) |  |
|  | *≥3 times /week* |  | 83 (47.70%) | 102 (43.00%) | 52 (48.10%) | 237 (45.70%) |  |
| **Eating eggs:** | *Never* |  | 18 (10.30%) | 44 (18.50%) | 19 (17.60%) | 81 (15.60%) | 0.091 |
|  | *1-2 times /week* |  | 98 (56.30%) | 112 (47.10%) | 60 (55.60%) | 270 (51.90%) |  |
|  | *≥3 times /week* |  | 58 (33.30%) | 82 (34.50%) | 29 (26.90%) | 169 (32.50%) |  |
| **Consumption of milk:** | *Never* |  | 70 (40.20%) | 112 (47.10%) | 32 (29.60%) | 214 (41.20%) | 0.039* |
|  | *1-2 times /week* |  | 49 (28.20%) | 66 (27.70%) | 39 (36.10%) | 154 (29.60%) |  |
|  | *≥3 times /week* |  | 55 (31.60%) | 60 (25.20%) | 37 (34.30%) | 152 (29.20%) |  |
| **Eating fried foods:** | *Never* |  | 41 (23.60%) | 54 (22.70%) | 11 (10.20%) | 106 (20.40%) | 0.046* |
|  | *1-2 times /week* |  | 81 (46.60%) | 102 (42.90%) | 56 (51.90%) | 239 (46.00%) |  |
|  | *≥3 times /week* |  | 52 (29.90%) | 82 (34.50%) | 41 (38.00%) | 175 (33.70%) |  |
| **Eating salty foods (bacon, salted fish, pickled mustard green, etc.):** | *Never* |  | 56 (32.20%) | 76 (31.90%) | 21 (19.40%) | 153 (29.40%) | 0.046* |
|  | *1-2 times /week* |  | 71 (40.80%) | 92 (38.70%) | 59 (54.60%) | 222 (42.70%) |  |
|  | *≥3 times /week* |  | 47 (27.00%) | 70 (29.40%) | 28 (25.90%) | 145 (27.90%) |  |
| **Eating spicy and or spiced foods:** | *Never* |  | 72 (41.40%) | 101 (42.40%) | 18 (16.70%) | 191 (36.70%) | <0.001* |
|  | *1-2 times /week* |  | 59 (33.90%) | 70 (29.40%) | 43 (39.80%) | 172 (33.10%) |  |
|  | *≥3 times /week* |  | 43 (24.70%) | 67 (28.20%) | 47 (43.50%) | 157 (30.20%) |  |
| **Consumption of sugars and sweets:** | *Never* |  | 24 (13.80%) | 32 (13.40%) | 8 (7.40%) | 64 (12.30%) | 0.124 |
|  | *1-2 times /week* |  | 78 (44.80%) | 96 (40.30%) | 39 (36.10%) | 213 (41.00%) |  |
|  | *≥3 times /week* |  | 72 (41.40%) | 110 (46.20%) | 61 (56.50%) | 243 (46.70%) |  |
| **Fish intake:** | *Never* |  | 39 (22.40%) | 85 (35.70%) | 27 (25.00%) | 151 (29.00%) | 0.022* |
|  | *1-2 times /week* |  | 119 (68.40%) | 133 (55.90%) | 75 (69.40%) | 327 (62.90%) |  |
|  | *≥3 times /week* |  | 16 (9.20%) | 20 (8.40%) | 6 (5.60%) | 42 (8.10%) |  |
| **Frozen meals intake:** | *Never* |  | 96 (55.20%) | 125 (52.50%) | 36 (33.30%) | 257 (49.40%) | 0.003* |
|  | *1-2 times /week* |  | 56 (32.20%) | 91 (38.20%) | 55 (50.90%) | 202 (38.80%) |  |
|  | *≥3 times /week* |  | 22 (1.60%) | 22 (9.20%) | 17 (15.70%) | 61 (11.70%) |  |
| **Vegetable intake:** | *Never* |  | 21 (12.10%) | 42 (17.60%) | 14 (13.00%) | 77 (14.80%) | 0.246 |
|  | *1-2 times /week* |  | 67 (38.50%) | 102 (42.90%) | 48 (44.40%) | 217 (41.70%) |  |
|  | *≥3 times /week* |  | 86 (49.40%) | 94 (39.50%) | 46 (42.60%) | 226 (43.50%) |  |
| **Consumption of fruits:** | *Never* |  | 27 (15.50%) | 38 (16.00%) | 15 (13.90%) | 80 (15.40%) | 0.353 |
|  | *1-2 times /week* |  | 77 (44.30%) | 117 (49.20%) | 61 (56.50%) | 255 (49.00%) |  |
|  | *≥3 times /week* |  | 70 (40.20%) | 83 (34.90%) | 32 (29.60%) | 185 (35.60%) |  |
| **Diet composition:** | *Vegetable-based* |  | 10 (5.70%) | 6 (2.50%) | 4 (3.70%) | 20 (3.80%) | 0.152 |
|  | *Mixed meals* |  | 162 (93.10%) | 221 (92.90%) | 101 (93.50%) | 484 (93.10%) |  |
|  | *Meat-based* |  | 2 (1.10%) | 11 (4.60%) | 3 (2.80%) | 16 (3.10%) |  |
| **Drinking water:** | *Tap water-based* |  | 117 (67.20%) | 136 (57.10%) | 64 (59.30%) | 317 (61.00%) | 0.108 |
|  | *Boiled water-based* |  | 1 (0.60%) | 5 (2.10%) | 0 (0.00%) | 6 (1.20%) |  |
|  | *Mineral water-based* |  | 56 (32.20%) | 97 (40.80%) | 44 (40.70%) | 197 (37.90%) |  |
| **Consumption of tea:** | *Yes* |  | 98 (56.30%) | 141 (59.20%) | 69 (63.90%) | 308 (59.20%) | 0.454 |
|  | *No* |  | 76 (43.70%) | 97 (40.80%) | 39 (36.10%) | 212 (40.80%) |  |
| **Frequency of tea consumption：** | *1-2 times /week* |  | 26 (26.50%) | 31 (22.00%) | 26 (37.70%) | 83 (26.90%) | 0.055 |
|  | *≥3 times /week* |  | 72 (73.50%) | 110 (78.00%) | 43 (62.30%) | 225 (73.10%) |  |
| **Main types of tea:** | *Black tea* |  | 76 (77.60%) | 111 (78.70%) | 53 (78.80%) | 240 (77.90%) | 0.996 |
|  | *Green tea* |  | 9 (9.20%) | 12 (8.50%) | 7 (10.10%) | 28 (9.10%) |  |
|  | *Scented tea* |  | 4 (4.10%) | 4 (2.80%) | 3 (4.30%) | 11 (3.60%) |  |
|  | *Others* |  | 9 (9.20%) | 14 (9.90%) | 6 (8.70%) | 29 (9.40%) |  |
| **Drinking Alcohol:** | *Yes* |  | 15 (8.60%) | 25 (10.50%) | 2 (1.90%) | 42 (8.10%) | 0.022* |
|  | *No* |  | 159 (91.40%) | 213 (89.50%) | 106 (98.10%) | 478 (91.90%) |  |
| **Frequency of drinking:** | *1-2 times /month* |  | 8 (53.30%) | 10 (40.00%) | 2 (100.00%) | 20 (47.60%) | 0.389 |
|  | *1-2 times /week* |  | 5 (33.30%) | 7 (28.00%) | 0 (0.00%) | 12 (28.60%) |  |
|  | *≥3 times/week* |  | 2 (13.30%) | 8 (32.00%) | 0 (0.00%) | 10 (23.8%) |  |
| **Type of alcohol:** | *White wine* |  | 0 (0.00%) | 4 (16.00%) | 0 (0.00%) | 4 (9.50%) | 0.037* |
|  | *Red wine* |  | 2 (13.30%) | 3 (12.00%) | 2 (100.00%) | 7 (16.70%) |  |
|  | *Beer* |  | 5 (33.30%) | 8 (32.00%) | 0 (0.00%) | 13 (31.00%) |  |
|  | *high alcohol spirits* |  | 8 (53.30%) | 10 (40.00%) | 0 (0.00%) | 18 (42.90%) |  |
| **Smoking:** | *Never smoking* |  | 120 (69.00%) | 165 (69.30%) | 81 (75.00%) | 366 (70.40%) | 0.497 |
|  | *Smoking* |  | 54 (31.00%) | 73 (30.70%) | 27 (25.00%) | 154 (29.60%) |  |
| **Type of smoking:** | *cigarettes* |  | 33 (61.10%) | 46 (63.00%) | 11 (40.70%) | 90 (58.40%) | 0.345 |
|  | *vaping* |  | 11 (20.40%) | 16 (21.90%) | 9 (33.30%) | 36 (23.40%) |  |
|  | *hookah (shisha)* |  | 10 (18.50%) | 11 (15.10%) | 7 (25.90%) | 28 (18.20%) |  |
| **Average number of cigarettes smoked or shisha (heads) per day:** | *<10 cigarettes* |  | 30 (55.60%) | 45 (61.60%) | 16 (59.30%) | 91 (59.10%) | 0.589 |
|  | *10-20 cigarettes* |  | 18 (33.30%) | 24 (32.90%) | 7 (25.90%) | 49 (31.80%) |  |
|  | *>20 cigarettes* |  | 6 (11.10%) | 4 (5.50%) | 4 (14.80%) | 14 (9.10%) |  |
| **If you are a current smoker or a former smoker - how long have you smoked?** | *<1year* |  | 5 (9.30%) | 7 (9.60%) | 2 (7.40%) | 14 (9.10%) | 0.563 |
|  | *1-5 years* |  | 11 (20.40%) | 25 (34.20%) | 7 (25.90%) | 43 (27.90%) |  |
|  | *5-10 years* |  | 12 (22.20%) | 11 (15.10%) | 3 (11.10%) | 26 (16.90%) |  |
|  | *≥10 years* |  | 26 (48.10%) | 30 (41.10%) | 15 (55.60%) | 71 (46.10%) |  |
| **Physical activity:** | *Never* |  | 55 (31.60%) | 85 (35.70%) | 32 (29.60%) | 172 (33.10%) | 0.499 |
|  | *1-2 times /week* |  | 77 (44.30%) | 87 (36.60%) | 44 (40.70%) | 208 (40.00%) |  |
|  | *≥3 times/week* |  | 42 (24.10%) | 66 (27.70%) | 32 (29.60%) | 140 (26.90%) |  |
| **Mean sleep duration:** | *＜6 hours* |  | 80 (46.00%) | 106 (44.50%) | 60 (55.60%) | 246 (47.30%) | 0.149 |
|  | *≥6 hours* |  | 94 (54.00%) | 132 (55.50%) | 48 (44.40%) | 274 (52.70%) |  |
| **Family history** | *Yes* |  | 38 (21.80%) | 54 (22.70%) | 16 (14.80%) | 108 (20.80%) | 0.225 |
|  | *No* |  | 136 (78.20%) | 184 (77.30%) | 92 (85.20%) | 412 (79.20%) |  |
| **Allergies:** | *Yes* |  | 41 (23.60%) | 92 (38.70%) | 34 (31.50%) | 167 (32.10%) | 0.005* |
|  | *No* |  | 133 (76.40%) | 146 (61.30%) | 74 (68.50%) | 353 (67.90%) |  |
| **Pet ownership:** | *Yes* |  | 48 (27.60%) | 60 (25.20%) | 32 (29.60%) | 140 (26.90%) | 0.672 |
|  | *No* |  | 126 (72.40%) | 178 (74.80%) | 76 (70.40%) | 380 (73.10%) |  |
| **Appendectomy:** | *Yes* |  | 11 (6.30%) | 46 (19.30%) | 3 (2.80%) | 60 (11.50%) | <0.001* |
|  | *No* |  | 163 (93.70%) | 192 (80.70%) | 105 (97.20%) | 460 (85.50%) |  |
| **Breast-feeding (I have been breast-fed before):** | *Never* |  | 37 (21.30%) | 45 (18.90%) | 23 (21.30%) | 105 (20.20%) | 0.622 |
|  | *﹤3 months* |  | 27 (15.50%) | 35 (14.70%) | 14 (13.00%) | 76 (14.60%) |  |
|  | *≥3 months* |  | 75 (43.10%) | 10 (43.70%) | 39 (36.10%) | 218 (41.90%) |  |
|  | *Unsure* |  | 3 (20.10%) | 54 (22.70%) | 32 (29.60%) | 121 (23.30%) |  |
| **Delivery mode:** | *Natural birth* |  | 151 (86.80%) | 200 (84.00%) | 99 (91.70%) | 450 (86.50%) | 0.155 |
|  | *Cesarean* |  | 23 (13.20%) | 38 (16.00%) | 9 (8.30%) | 70 (13.50%) |  |
| **Childhood antibiotic use (before 14 years):** | *Never* |  | 41 (23.60%) | 40 (16.80%) | 30 (27.80%) | 111 (21.30%) | 0.011* |
|  | *1-2 times /year* |  | 45 (25.90%) | 52 (21.80%) | 22 (20.40%) | 119 (22.90%) |  |
|  | *≥3 times/year* |  | 24 (13.80%) | 53 (22.30%) | 9 (8.30%) | 86 (16.50%) |  |
|  | *Unsure* |  | 64 (36.80%) | 93 (39.10%) | 47 (43.50%) | 204 (39.20%) |  |
| **Childhood gastrointestinal infections (before 14 years):** | *Never* |  | 98 (56.30%) | 118 (49.60%) | 67 (62.00%) | 283 (54.40%) | 0.098 |
|  | *1-2 times /year* |  | 18 (10.30%) | 26 (10.90%) | 7 (6.50%) | 51 (9.80%) |  |
|  | *≥3 times/year* |  | 14 (8.00%) | 34 (14.30%) | 6 (5.60%) | 54 (10.40%) |  |
|  | *Unsure* |  | 44 (25.30%) | 60 (25.20%) | 28 (25.90%) | 132 (25.40%) |  |
| **Non-aspirin non-steroidal anti-inflammatory drugs (NA-NSAIDs) intake** | *Never* |  | 98 (56.30%) | 159 (66.80%) | 73 (67.60%) | 330 (63.50%) | 0.054* |
|  | *＜1 month* |  | 43 (24.70%) | 54 (22.70%) | 25 (23.10%) | 122 (23.50%) |  |
|  | *≥1 month* |  | 33 (19.00%) | 25 (10.50%) | 10 (9.30%) | 68 (13.10%) |  |
| **Aspirin intake:** | *Never* |  | 147 (84.50%) | 211 (88.70%) | 95 (88.00%) | 453 (87.10%) | 0.316 |
|  | *＜1 month* |  | 20 (11.50%) | 18 (7.60%) | 6 (5.60%) | 44 (8.50%) |  |
|  | *≥1 month* |  | 7 (4.00%) | 9 (3.80%) | 7 (6.50%) | 23 (4.40%) |  |
| **Oral contraceptive use:** | *Never* |  | 91 (52.60%) | 148 (62.20%) | 77 (71.30%) | 316 (60.90%) | 0.046* |
|  | *Past* |  | 27 (15.60%) | 38 (16.00%) | 14 (13.00%) | 79 (15.20%) |  |
|  | *Current,＜5 years* |  | 13 (7.50%) | 7 (2.90%) | 2 (1.90%) | 22 (4.20%) |  |
|  | *Current,≥5 years* |  | 7 (4.00%) | 8 (3.40%) | 1 (0.90%) | 16 (3.10%) |  |
|  | *Not applicable* |  | 35 (20.20%) | 37 (15.50%) | 14 (13.00%) | 86 (16.60%) |  |
| **Parasitic infection:** | *Never* |  | 119 (68.40%) | 140 (58.80%) | 86 (79.60%) | 345 (66.30%) | 0.002* |
|  | *Past* |  | 29 (16.70%) | 43 (18.10%) | 7 (6.50%) | 79 (15.20%) |  |
|  | *Unsure* |  | 26 (14.90%) | 55 (23.10%) | 15 (13.90%) | 96 (18.50%) |  |
