# Supplementary material for: Modifiable risk factors for inflammatory bowel disease in Kuwait: A cross-sectional analysis
Source: PLoS One. 2025 Dec 2;20(12):e0338005. doi: 10.1371/journal.pone.0338005 (PMC12671769; doi:10.1371/journal.pone.0338005)
Supplement: S3 Table — (DOCX) [file pone.0338005.s003.docx]

**Table 3. Crude odds ratios for factors associated with IBD (unadjusted univariable logistic regression)**

| **Variable:** | **Reference Category** | **OR Exp(B)** | **95% C.I.for EXP(B)** |  | **p-value** |
| --- | --- | --- | --- | --- | --- |
|  |  |  | **Lower** | **Upper** |  |
| ***Sex:*** | *Female* | 0.548 | 0.338 | 0.89 | 0.015* |
| ***Age:*** |  | 0.984 | 0.966 | 1.003 | 0.106 |
| ***Occupational Factors:*** |  |  |  |  |  |
| ***Work type:*** | *Manual* |  |  |  | 0.001* |
| *Mental vs. manual* |  | 1.932 | 0.822 | 4.54 | 0.091 |
| *Mixed vs. manual* |  | 2.342 | 1.46 | 3.755 | p<0.001 |
| ***Work Stress*** | *No stress* |  |  |  | 0.027* |
| *Mild stress* |  | 1.926 | 0.756 | 4.906 | 0.169 |
| *Moderate stress* |  | 3.551 | 1.317 | 9.571 | 0.012* |
| *Much stress* |  | 2.259 | 1.071 | 4.766 | 0.032* |
| *Extreme stress* |  | 1.288 | 0.601 | 2.76 | 0.515 |
| ***Dietary Patterns:*** |  |  |  |  |  |
| ***irregular mealtime*** | *Never* | 1.431 |  |  | 0.489 |
| *1-2 times/week:* |  | 1.359 | 0.732 | 2.523 | 0.331 |
| *≥3 times/week:* |  | 0.923 | 0.58 | 1.47 | 0.736 |
| ***Meat intake*** | *Never* |  |  |  | 0.412 |
| *1-2 times/week:* |  | 1.722 | 0.767 | 3.863 | 0.188 |
| *≥3 times/week:* |  | 1.036 | 0.665 | 1.614 | 0.874 |
| ***eating salty foods*** | *Never* |  |  |  | 0.011* |
| *1-2 times/week:* |  | 1.504 | 0.811 | 2.791 | 0.195 |
| *≥3 times/week:* |  | 0.661 | 0.398 | 1.1 | 0.111 |
| ***fish intake:*** | *Never* |  |  |  | 0.254 |
| *1-2 times/week:* |  | 0.765 | 0.293 | 1.998 | 0.585 |
| *≥3 times/week:* |  | 0.56 | 0.227 | 1.38 | 0.208 |
| ***consumption of sugar/sweets*** | *Never* |  |  |  | 0.048* |
| *1-2 times/week:* |  | 2.346 | 1.059 | 5.198 | 0.036 |
| *≥3 times/week:* |  | 1.495 | 0.951 | 2.351 | 0.081 |
| ***Consumption of milk*** | *Never* |  |  |  |  |
| *1-2 times/week:* |  | 1.83 | 1.08 | 3.101 | 0.025* |
| *≥3 times/week:* |  | 0.949 | 0.565 | 1.594 | 0.842 |
| ***Eating fried foods*** | *Never* |  |  |  | 0.016* |
| *1-2 times/week:* |  | 2.642 | 1.292 | 5.405 | 0.008* |
| *≥3 times/week:* |  | 1.313 | 0.631 | 1.584 | 0.244 |
| ***Eating spicy foods*** | *Never* |  |  |  | p<0.001* |
| *1-2 times/week:* |  | 4.107 | 2.268 | 7.434 | p<0.001* |
| *≥3 times/week:* |  | 1.282 | 0.789 | 2.083 | 0.316 |
| ***Frozen meal intake*** | *Never* |  |  |  | 0.001* |
| *1-2 times/week:* |  | 2.372 | 1.224 | 4.595 | 0.010* |
| *≥3 times/week:* |  | 1.033 | 0.545 | 1.958 | 0.922 |
| ***vegetable intake:*** | *Never* |  |  |  | 0.755 |
| *1-2 times/week:* |  | 1.15 | 0.592 | 2.233 | 0.686 |
| *≥3 times/week:* |  | 0.9 | 0.57 | 1.419 | 0.655 |
| ***consumption of fruits*** |  |  |  |  | 0.215 |
| *1-2 times/week:* | *Never* | 0.906 | 0.46 | 1.786 | 0.776 |
| *≥3 times/week:* |  | 0.665 | 0.413 | 1.072 | 0.094 |
| ***diet comptosition*** | *Vegetable-based* |  |  |  | 0.975 |
| *mixed vs. vegetable-based* |  | 0.923 | 0.174 | 4.885 | 0.925 |
| *meat vs. vegetable-based* |  | 0.875 | 0.245 | 3.13 | 0.837 |
| ***Drinking Alcohol*** | *Yes* | 5.699 | 1.355 | 23.968 | 0.018* |
| ***Smoking*** | *Yes* | 0.748 | 0.461 | 1.213 | 0.239 |
| ***Physical activity*** | *Never* |  |  |  | 0.645 |
| *1-2 times/week:* |  | 1.296 | 0.747 | 2.248 | 0.356 |
| *≥3 times/week:* |  | 1.104 | 0.659 | 1.85 | 0.706 |
| ***Family history*** | *Yes* | 1.653 | 0.926 | 2.951 | 0.089 |
| ***Allergies*** | *Yes* | 1.038 | 0.658 | 1.636 | 0.874 |
| ***Pet ownership*** | *Yes* | 0.844 | 0.529 | 1.347 | 0.477 |
| ***Breastfeeding*** | *Never* |  |  |  | 0.287 |
| *<3 months* |  | 1.282 | 0.694 | 2.369 | 0.428 |
| *≥3 months* |  | 1.592 | 0.785 | 3.228 | 0.197 |
| *unsure* |  | 1.65 | 0.969 | 2.81 | 0.065 |
| ***Delivery mode*** | *Natural* | 0.523 | 0.251 | 1.09 | 0.084 |
| ***Mean sleep duration:*** | *<6 hours* | 0.658 | 0.43 | 1.008 | 0.055* |
| ***Appendectomy*** | *Yes* | 5.62 | 1.725 | 18.311 | 0.004* |
| ***Childhood antibiotic use (before 14 years)*** | *Never* |  |  |  | 0.033* |
| *1-2 times/year* |  | 1.32 | 0.749 | 2.325 | 0.337 |
| *≥3 times/year* |  | 2.561 | 1.94 | 5.496 | 0.016* |
| ***Childhood gastrointestinal infections (before 14 years):*** | *Never* |  |  |  | 0.121 |
| *1-2 times/year* |  | 0.868 | 0.527 | 1.43 | 0.578 |
| *≥3 times/year* |  | 1.692 | 0.688 | 4.163 | 0.252 |
| *unsure* |  | 2.154 | 0.837 | 5.545 | 0.112 |
| ***Non-aspirin non-steroidal anti-inflammatory drugs (NA-NSAIDs) intake*** | *Never* |  |  |  | 0.394 |
| <1 month |  | 0.607 | 0.296 | 1.247 | 0.174 |
| ≥1 month |  | 0.669 | 0.3 | 1.492 | 0.326 |
| ***Aspirin intake*** | *Never* |  |  |  | 0.271 |
| *<1 month* |  | 1.649 | 0.659 | 4.123 | 0.285 |
| *≥1 month* |  | 2.771 | 0.804 | 9.546 | 0.106 |
| ***Oral contraceptive use*** | *Never* |  |  |  | 0.121 |
| *past* |  | 0.604 | 0.322 | 1.13 | 0.115 |
| *current, <5years* |  | 0.903 | 0.4 | 2.036 | 0.805 |
| *current, ≥5years* |  | 1.944 | 0.408 | 9.274 | 0.404 |
| *not applicable* |  | 2.917 | 0.356 | 23.905 | 0.319 |
| **Parasitic infection** | *Never* |  |  |  | 0.004* |
| *past* |  | 0.558 | 0.305 | 1.019 | 0.058 |
| *unsure* |  | 1.905 | 0.735 | 4.933 | 0.184 |
