# Supplementary material for: Modifiable risk factors for inflammatory bowel disease in Kuwait: A cross-sectional analysis
Source: PLoS One. 2025 Dec 2;20(12):e0338005. doi: 10.1371/journal.pone.0338005 (PMC12671769; doi:10.1371/journal.pone.0338005)
Supplement: S4 Table — (DOCX) [file pone.0338005.s004.docx]

**Table 4. Adjusted ORs and p-values for factors associated with IBD in the multivariable logistic regression model**

| Variable | Reference Category | Adjusted OR | 95% CI | p-value |
| --- | --- | --- | --- | --- |
| Sex | Male vs. Female (ref) | 2.487 | 1.315 – 4.704 | 0.005* |
| Age | Per 1 year increase | 0.964 | 0.940 – 0.988 | 0.004* |
| Work type |  |  |  |  |
|  | Mental vs Manual (ref) | 1.818 | 1.032 – 3.204 | 0.039* |
|  | Mixed vs Manual (ref) | 1.362 | 0.467 – 3.974 | 0.572 |
| Work stress |  |  |  |  |
|  | Mild vs No stress (ref) | 1.488 | 0.456 – 4.857 | 0.511 |
|  | Moderate vs No stress (ref) | 1.811 | 0.552 – 5.936 | 0.327 |
|  | Severe vs No stress (ref) | 1.565 | 0.609 – 4.020 | 0.353 |
| Dietary patterns: |  |  |  |  |
| Consumption of milk |  |  |  |  |
|  | 1–2x/week vs Never (ref) | 1.557 | 0.816 – 2.972 | 0.188 |
|  | ≥3x/week vs Never (ref) | 1.216 | 0.656 – 2.255 | 0.535 |
| Eating fried foods |  |  |  |  |
|  | 1–2x/week vs Never (ref) | 1.283 | 0.516 – 3.189 | 0.591 |
|  | ≥3x/week vs Never (ref) | 0.747 | 0.415 – 1.344 | 0.333 |
| Eating spicy foods |  |  |  |  |
|  | 1–2x/week vs Never (ref) | 2.631 | 1.287 – 5.378 | 0.008* |
|  | ≥3x/week vs Never (ref) | 1.054 | 0.585 – 1.900 | 0.866 |
| Consumption of sugar/sweets |  |  |  |  |
|  | 1–2x/week vs Never (ref) | 1.508 | 0.552 – 4.116 | 0.423 |
|  | ≥3x/week vs Never (ref) | 1.688 | 0.973 – 2.928 | 0.062 |
| Frozen meal intake |  |  |  |  |
|  | 1–2x/week vs Never (ref) | 1.686 | 0.751 – 3.787 | 0.206 |
|  | ≥3x/week vs Never (ref) | 0.904 | 0.420 – 1.946 | 0.797 |
| Drinking alcohol | Yes vs No (ref) | 6.508 | 1.418 – 29.863 | 0.016* |
| Mean sleep duration | >6h vs <6h (ref) | 0.727 | 0.437 – 1.209 | 0.219 |
| Family history | Yes vs No (ref) | 2.141 | 1.109 – 4.134 | 0.023* |
| Appendectomy | Yes vs No (ref) | 7.158 | 3.095 – 16.582 | 0.003* |
| Delivery mode | Cesarean vs Natural birth (ref) | 0.716 | 0.309 – 1.658 | 0.435 |
| Oral contraceptive use |  |  |  |  |
|  | Past vs Never (ref) | 0.692 | 0.320 – 1.493 | 0.343 |
|  | Current <5y vs Never (ref) | 1.784 | 0.630 – 5.051 | 0.275 |
|  | Current ≥5y vs Never (ref) | 3.185 | 0.658 – 22.683 | 0.135 |
|  | Not applicable vs Never (ref) | 8.187 | 0.834 – 80.323 | 0.071 |
| Parasitic infection |  |  |  |  |
|  | Past vs Never (ref) | 0.484 | 0.240 – 0.973 | 0.042* |
|  | Current vs Never (ref) | 1.748 | 0.609 – 5.021 | 0.304 |
