# Supplementary material for: Modifiable risk factors for inflammatory bowel disease in Kuwait: A cross-sectional analysis
Source: PLoS One. 2025 Dec 2;20(12):e0338005. doi: 10.1371/journal.pone.0338005 (PMC12671769; doi:10.1371/journal.pone.0338005)
Supplement: S5 Table — (DOCX) [file pone.0338005.s005.docx]

**Table 5. Stepwise logistic regression models showing the impact of covariate adjustment on the association between selected exposures and IBD status:**

| ***Exposure Variable*** | ***Crude Odds Ratio (95% CI)*** | ***Model 2: Adjusted for Sex (95% CI)*** | ***Model 3: Adjusted for Sex + Age (95% CI)*** |
| --- | --- | --- | --- |
| *Sex (male vs. female)* | 0.548 (0.338-0.89) | -- | 2.487 (1.315 – 4.704) |
| *Age (per year increase)* | 0.984 (0.966-1.003) | 0.980 (0.961-1.00) | 0.964 (0.940 – 0.988) |
| *Work type (Mental vs. Manual)* | 1.932 (0.822-4.54) | 1.958 (0.830-4.619) | 1.818 (1.032 – 3.204) |
| *Alcohol consumption (No vs. Yes)* | 5.699 (1.355-23.968) | 5.551 (1.317-23.426) | 6.508 (1.418 – 29.863) |
| *Eating Spicy Food 1-2times/week vs. never)* | 4.107 (2.268-7.434) | 4.055 (2.234-7.359) | 2.631 (1.287 – 5.378) |
| *Family history (No vs. Yes)* | 1.653 (0.926-2.951) | 1.729 (0.965-3.097) | 2.141 (1.109 – 4.134) |
| *Parasitic infection (Past vs. never)* | 0.558 (0.305-1.019) | 0.547 (0.299-1.002) | 0.484 (0.240 – 0.973) |
| *Appendectomy (No vs. Yes)* | 5.62 (1.725-18.311) | 5.913 (1.810-19.319) | 7.158 (3.095 – 16.582) |
