# Supplementary material for: Modifiable risk factors for inflammatory bowel disease in Kuwait: A cross-sectional analysis
Source: PLoS One. 2025 Dec 2;20(12):e0338005. doi: 10.1371/journal.pone.0338005 (PMC12671769; doi:10.1371/journal.pone.0338005)
Supplement: S1 File — English version of the questionnaire assessing environmental and lifestyle factors associated with IBD. (DOCX) [file pone.0338005.s006.docx]

**S1. File Questionnaire in English**

**Number:** **Date:**

[**Hospital**](http://cn.bing.com/dict/clientsearch?mkt=zh-CN&setLang=zh&form=BDVEHC&ClientVer=BDDTV3.5.0.4311&q=%E7%A1%AE%E8%AF%8A%E5%8C%BB%E9%99%A2) **for** [**diagnosis**](http://cn.bing.com/dict/clientsearch?mkt=zh-CN&setLang=zh&form=BDVEHC&ClientVer=BDDTV3.5.0.4311&q=%E7%A1%AE%E8%AF%8A%E5%8C%BB%E9%99%A2)**:**

**Diagnosis:**

口Ulcerative colitis (UC) 口Crohn’s disease (CD)

**Year diagnosed with disease** (……)

**Please fill in the following questions:**

Do you have any other significant health issues or medical conditions not outlined or

mentioned on this form? *(Complete additional questionnaires, as indicated)*

口 Condition(s) List treatment and current status

口 NO other medical conditions or health issues.

**Please fill in some basic information:**

1. **Name:**
2. **Sex:** 口 Male 口 Female
3. **Age:** (yrs.)
4. **Height:**  (cm)
5. **Weight:**  (kg)
6. **Nationality:**

**If you are a foreigner, please specify how many years you have lived in Kuwait (_____)**

1. **Education level:** 口Primary school 口Secondary school 口University
2. **Occupational type:** 口 governmental employee 口 private sector employee 口 free lancers

口 student 口 retired 口 Others **^………….^**

14. **Marital status:** 口 Unmarried口Married口Divorced口Widowed

15. **Please fill in some items about working and living environment:**

15.1 **Work type:** 口 Manual work 口 Mental work 口 Mixed work

*(**People who do manual work include unskilled manual workers, like construction workers, as well as farm and forestry workers. People who do mental work include desk jobs, like clerical workers, managers, higher administrators, scientific research-related workers, and clerical employees. Mixed work includes some skilled and specialized work required more manual work, like dentists, drivers, seamen, cooks.)*

15.2 **Work stress:**  口 no stress 口 mild stress 口 moderate stress

口 much stress 口 extreme stress

**Some eating habits and lifestyle prior to diagnosis:**

1. **Irregular mealtimes:** 口 Never 口1-2 times /week 口≥3 times /week

2. **Eating meat:** 口 Never 口1-2 times /week 口≥3 times /week

3. **Eating eggs:** 口 Never 口1-2 times /week 口≥3 times /week

4. **Consumption of milk:** 口 Never 口1-2 times /week 口≥3 times /week

5. **Eating fried foods:** 口 Never 口1-2 times /week 口≥3 times /week

6. **Eating salty foods** *(bacon, salted fish, pickled mustard green, etc.)***:**

口 Never 口1-2 times /week 口≥3 times /week

7. **Eating spicy and or spiced foods:** 口 Never 口1-2 times /week 口≥3 times /week

8. **Consumption of sugars and sweets:**

口 Never 口1-2 times /week 口≥3 times /week

9. **Fish intake:** 口Never口1-2 times /week 口≥3 times /week

10. **Frozen dinners intake:** 口Never口1-2 times /week口≥3 times /week

11. **Vegetable intake:** 口Never口1-2 times /week口≥3 times /week

12. **Consumption of fruits:** 口Never口1-2 times /week口≥3 times /week

13. **Drinking water:** 口 Tap water-based 口 Boiled water-based 口 Mineral water-based

14. **Diet composition:** 口 Vegetable-based 口 Mixed meals 口 Meat-based

15. **Consumption of tea** *(If no, skip this item; If yes, please fill in 15.1and 15.2)***:**

口 No 口 Yes

15.1 **Frequency of tea consumption：**口1-2 times /week口≥3 times /week

15.2 **Main types of tea:** 口 Black tea 口 Green tea 口 Scented tea 口 Others,

*(Green tea is pale, greenish yellow, and the black tea is a deep amber color.)*

16. **Smoking** *(If never, skip this item; If current, please fill in 16.1and 16.2)***:**

口 Never smoking 口 Current smoking 口 Ex- smoking: ………………………

**Type of smoking:** 口 cigarettes 口 vaping 口 hookah (shisha) 口 pipe smokers

16.1 **Average number of cigarettes smoked per day:**

口<10 cigarettes 口10-20 cigarettes 口>20 cigarettes

16.2 **Duration of smoking:**

口<1year口1-5 years口5-10 years口≥10 years

*(Current smoking was defined as smoking at least one cigarette/day, ex-smoking as reporting having smoked at least one cigarette/day but having quit, and never smoking as never having smoked one cigarette/day.)*

17. **Drinking Alcohol** *(If no, skip this item; If yes, please fill in 17.1, 17.2 and 17.3*):

口No 口Yes

*(Drinking is defined as more than once of alcohol drinking per month.)*

17.1 **Frequency of drinking:**

口1-2 times /month 口1-2 times /week 口≥3 times/week

17.2 **Type of alcohol:**

口 White wine 口 Red wine 口 Beer 口 high alcohol spirits

18. **Physical activity:**

口 Never 口1-2 times /week 口≥3 times/week

*(Physical activity is any rhythmic and continuous activity more than 20 minutes at a time).*

19. **Mean sleep duration:** 口＜6 hours口≥6 hours

**Other items prior to the illness onset:**

1. **Family history** *(parent or siblings had Ulcerative colitis or Crohn’s disease)***:**

口 No 口 Yes

2. **Allergies:** 口 No 口 Yes,

3. **Pet ownership:** 口 No 口 Yes,

4. **Appendectomy:** 口 No 口 Yes

5. **Breast-feeding (I have been breastfed before):**

口 Never 口﹤3 months 口≥3 months 口 Unsure

6. **Delivery mode:** 口 Natural birth 口 Cesarean

7. **Childhood antibiotic use (before 14 years):**

口 Never 口 1-2 times /year 口 ≥3 times/year 口 Unsure

8. **Childhood gastrointestinal infections (before 14 years):**

口 Never 口 1-2 times /year 口 ≥3 times/year 口 Unsure

10. **Non-aspirin non-steroidal anti-inflammatory drugs (NA-NSAIDs) intake** (*ibuprofen, diclofenac, etc.)***:**

口 Never 口＜1 month 口≥1 month

11. **Aspirin intake:** 口 Never 口 ＜1 month 口 ≥1 month

12. **Oral contraceptive use:** 口 Never 口 Past 口Current,＜5 years 口Current,≥5 years

13. **Parasitic infection:**口 Never 口 Past 口 Uns
