# Supplementary material for: Modifiable risk factors for inflammatory bowel disease in Kuwait: A cross-sectional analysis
Source: PLoS One. 2025 Dec 2;20(12):e0338005. doi: 10.1371/journal.pone.0338005 (PMC12671769; doi:10.1371/journal.pone.0338005)
Supplement: S2 File — Arabic version of the questionnaire assessing environmental and lifestyle factors associated with IBD. (DOCX) [file pone.0338005.s007.docx]

**الرقم:** ــــــــــــــــ **التاريخ:** ــــــــــــــــــــ

**مستشفى التشخيص**: ــــــــــــــــــــــــــــــــــ

**التشخيص**:

التهاب القولون التقرحي 口

مرض كرون 口

في أي سنة تم تشخيص المرض: ..........

**يرجى ملء الأسئلة التالية:**

هل لديك أي مشاكل صحية مهمة أخرى أو حالات طبية غير مذكورة

في هذا النموذج؟

(أكمل الاستبيانات الإضافية، كما هو محدد)

الحالة (الحالات):

قائمة العلاج والوضع الحالي:

口 لا توجد حالات طبية أو مشاكل صحية أخرى.

**يرجى ملء بعض المعلومات الأساسية:**

٢. **الجنس:**

口ذكر

口انثى

٣. **العمر (بالسنوات)**:

٤. **الطول (سم):**

٥. **الوزن (كلغ):**

٩**. الجنسية:**

في حال ان كنت مقيماً في دولة الكويت، يرجى تحديد كم سنة عشتها في دولة الكويت (ـــــــ)

١٠**. مستوى التعليم:**

口ابتدائي

口 ثانوي

口 جامعي

١١. **نوع المهنة:**

口 عمل مستقل 口 عمال口 فلاحون 口محترفون※

口 الموظفون العموميون 口 ا آخرون※※.

*(※ يشمل المهنيون المدرسين والأطباء والممرضات وما إلى ذلك. ※※ يشمل الآخرون ربات البيوت والمتقاعدين والأشخاص المنتظرين للوظائف والعاملين بالعمالة الجزئية، إلخ.)*

١٢. **الحالة الاجتماعية:**

口 أعزب

口 متزوج

口 مطلق

口 أرمل

١٣. **يرجى ملء بعض البنود الخاصة ببيئة العمل والمعيشة:**

١٣.١ **نوع العمل:**

عمل يدوي 口

عمل عقلي 口

عمل عقلي يدوي 口

*(يشمل الأشخاص الذين يقومون بالأعمال اليدوية العمال اليدويين غير المهرة، مثل عمال البناء، وكذلك عمال المزارع والحراجة. ويشمل الأشخاص الذين يقومون بعمل عقلي الوظائف المكتبية، مثل العمال الكتابيين والمدراء والمسؤولين الأعلى والعاملين في مجال البحث العلمي والموظفين الكتابيين تشمل العمالة المختلطة بعض الأعمال الماهرة والمتخصصة التي تتطلب عمالة يدوية أكثر، مثل أطباء الأسنان، والسائقين، والبحارة، والطهاة.)*

١٣.٢ **ضغوط العمل:**

لا، أبدا. 口

معتدل. 口

ضغط عمل عالي 口

١٤. **بعض عادات الأكل ونمط الحياة (قبل تشخيص المرض):**

١. **أوقات وجبات غير منظمة:**

لا口

١-٢ / أسبوع 口

≥ ٣ مرات / أسبوع口

٢. **تناول اللحوم:**

لا 口

١-٢ مرات في الأسبوع 口

≥ ٣ مرات / أسبوع 口

٣. **تناول البيض:**

لا口

١-٢ / أسبوع 口

≥ ٣ مرات / أسبوع口

٤. **استهلاك الحليب:**

لا 口

١-٢/ أسبوع 口

≥ ٣ مرات / أسبوع口

٥.**تناول الأطعمة المقلية:**

لا口

١-٢/ أسبوع 口

≥ ٣ مرات / أسبوع口

٦. **تناول الأطعمة المالحة:**

*(اللحم المقدد، الأسماك المملحة، المخللات والخردل، ما إلى ذلك.)*

لا 口

١-٢/ أسبوع 口

≥ ٣ مرات / أسبوع口

٧. **تناول الأطعمة الحارة والمُبهرة:**

لا口

١-٢/ أسبوع口

≥ ٣ مرات / أسبوع口

٨. **استهلاك السكريات والحلويات:**

لا 口

١-٢/ أسبوع口

≥ ٣ مرات / أسبوع口

٩. **تناول الأسماك:**

لا口

١-٢/ أسبوع口

≥ ٣ مرات / أسبوع口

١٠. **تناول الوجبات المثلجة الجاهزة:**

لا口

١-٢/ أسبوع口

≥ ٣ مرات / أسبوع口

١١. **تناول الخضراوات:**

لا 口

١-٢/ أسبوع口

≥ ٣ مرات / أسبوع口

١٢. **تناول الفاكهة:**

لا口

١-٢/ أسبوع口

≥ ٣ مرات / أسبوع口

١٣. **النظام الغذائي:**

نباتي 口

قائم على اللحوم فقط口

مختلط 口

١٤. **استهلاك الشاي: (إذا كانت الإجابة لا، تخطي هذا السؤال؛ إذا كانت الإجابة نعم، يرجى ملء١٤.١، ١٤.٢):**

نعم 口

لا 口

١٤.١ **مرات شرب الشاي:**

١-٢/ أسبوع口

≥ ٣ مرات / أسبوع口

١٤.٢ **نوع الشاي:**

口 شاي أسود

口 شاي أخضر

口 أنواع أخرى

١٥. **التدخين: (إذا الإجابة لا، تخطي هذا السؤال، إذا كانت الإجابة نعم حاليًا أو سابقا، يرجى ملء ١٥.١ و١٥.٢):**

口 لا، أبدا.

口 مدحن حالي

口 مدخن سابق

نوع التدخين:

سجائر

تدخين الكتروني

شيشة

١٥.١ **متوسط عدد السجائر او احجار الشيشة التي يتم تدخينها يوميا:**

口 < ٢٠ سيجارة يوميا او حجر شيشة واحد يوميا

口 ١٠-٢٠ سيجارة يوميا ٢-٣ حجر شيشة يوميا

口 > أكثر من ٢٠ سيجارة يوميا، اكثر من ٣ حجر شيشة يوميا

١٥.٢ **مدة التدخين:**

口 < أقل من سنة

口 ١-٥ سنوات

口٥-١٠ سنوات

口 > أكثر من ١٠ سنوات

١٦. **شرب الكحول: (إذا كانت الإجابة لا، تخطي هذا السؤال، إذا كانت الإجابة نعم، فيرجى ملء ١٦.١ و١٦.٢ و١٦.٣):**

口 نعم

口 لا

١٦.١ **عدد مرات شرب الكحول:**

口 ١-٢ مرة/شهر

口 ١-٢ مرة/اسبوع

口 ≥ ٣ مرات / أسبوع

١٦.٢ **نوع الكحول:**

口 نبيذ أبيض

口 نبيذ أحمر

口 بيرة

口 مشروبا روحية عالية الكحول

١٧. **النشاط البدني:**

口 لا

١-٢ مرة/ أسبوع 口

≥ ٣ مرات / أسبوع口

*(النشاط البدني هو أي نشاط مستمر أكثر من 20 دقيقة في المرة الواحدة).*

١٨. **متوسط مدة النوم:**

口 > ٦ ساعات

≥口 ٦ ساعات

**أسئلة أخرى (يرجى الرد وفقا للحالة قبل تشخيص المرض):**

١.**التاريخ العائلي (إصابة أحد الوالدين أو الأشقاء أو الأقارب بالتهاب القولون التقرحي أو مرض كرون):**

口 نعم

口 لا

٢. **أي نوع من أنواع الحساسية:**

口 نعم

口 لا

٣. **ملكية أي حيوان أليف:**

口 نعم

口 لا

٤. **استئصال الزائدة الدودية:**

口 نعم

口 لا

٥. **الرضاعة الطبيعية (هل تم إرضاعك طبيعيا؟):**

口 لا

口 أقل من ٣ شهور

口 أكثر من ٣ شهور

口 متردد/ لا أعلم

٦. **طريقة الولادة (كيف تمت ولادتك؟):**

口 طبيعية

口 ولادة قيصرية

口 لا أعلم

٧. **استخدام المضادات الحيوية في سن الطفولة (قبل ١٤ سنة):**

口 نعم

口 لا

口 متردد/ لا أعلم

٨. **التهابات الجهاز الهضمي في فترة الطفولة:**

口 لا

口١-٢ مرة/ سنة

口 ٣ أو أكثر / سنة

口 متردد/ لا أعلم

١٠. **تناول العقاقير غير الستيرويدية المضادة للالتهابات التي لا تحتوي على الأسبرين (إيبوبروفين، ديكلوفيناك، إلخ):**

口 لا

口 مرة خلال الشهر الواحد

口 أكثر من مرة خلال الشهر الواحد

١١. **تناول الأسبرين:**

口 لا

口 مرة خلال الشهر الواحد

口 أكثر من مرة خلال الشهر الواحد

١٢. **استخدام وسائل منع الحمل (عن طريق الفم):**

口 لا

口 في الماضي

口 يؤخذ حاليا، أكثر من ٥ سنوات

口 يؤخذ حاليا، أقل من ٥ سنوات

١٣. **أي عدوى طفيلية:**

口 نعم في الماضي

口 لا

口 متردد/ لا أعلم
